# SLC15A3-mediated dipeptide metabolism confers antimetabolite resistance in lymphoma via mTORC1 activation

**DOI:** 10.1172/JCI199709

**Published:** 2026-07-15

**Authors:** Haojun Yang, Vincenzo Andrea Zingaro, Kevin Boardman, Ashish Noronha, Ekin Guney, Lingru Xue, Saishma Hoigebazar, Isabelle Liu, Sohit Miglani, Siyu Chen, Hieu Vu, Kwun Wah Wen, Hao G. Nguyen, Hani Goodarzi, Ralph J. DeBerardinis, Davide Ruggero

**Affiliations:** 1 Helen Diller Family Comprehensive Cancer Center, and; 2 Department of Urology, School of Medicine, UCSF, San Francisco, California, USA.; 3 Department of Pharmacological Sciences, School of Medicine, Stony Brook University, Stony Brook, New York, USA.; 4 Department of Pathology,; 5 Department of Neurology, and; 6 Department of Biochemistry & Biophysics, UCSF, San Francisco, California, USA.; 7 Arc Institute, Palo Alto, California, USA.; 8 Department of Pathology and Center of Excellence for Leukemia Studies, St. Jude Children’s Research Hospital, Memphis, Tennessee, USA.; 9 Eugene McDermott Center for Human Growth and Development, Children’s Research Institute, Department of Pediatrics, and; 10 Howard Hughes Medical Institute, University of Texas Southwestern Medical Center, Dallas, Texas, USA.; 11 Department of Cellular and Molecular Pharmacology, UCSF, San Francisco, California, USA.

**Keywords:** Hematology, Metabolism, Oncology, Drug therapy, Lymphomas

## Abstract

Antimetabolites, chemotherapy targeting nucleotide biosynthesis, are among the oldest and most widely used cancer treatments, yet resistance remains a daunting barrier, especially in the fight against B cell lymphomas. However, the underlying mechanisms of this resistance have long remained elusive. Using an innovative, integrated omics approach, we unexpectedly identified that the accumulation of dipeptides and upregulation of the dipeptide transporter SLC15A3 underlie resistance to nucleotide deficiency in a *Myc*-driven large B cell lymphoma mouse model. A similar mechanism occurred after long treatment of human B cell lymphoma cells with the chemotherapeutic purine synthesis inhibitor 6-mercaptopurine (6MP). Mechanistically, we demonstrated that dipeptides containing essential amino acids activated the growth and survival mTOR complex 1 (mTORC1) signaling pathway. Notably, SLC15A3 specifically interacted with mTOR on the lysosome, boosting mTORC1 activity selectively in resistant lymphoma cells but not in parental cancer cells. Silencing SLC15A3 diminished mTORC1 activity and restored resistant lymphoma sensitivity to 6MP. Strikingly, resistant lymphomas, but not primary tumors, exhibited heightened sensitivity to the clinical mTOR inhibitor, rapamycin, in culture and in vivo. We extended these findings in human lymphoma biopsies, which revealed increased SLC15A3 expression following antimetabolite therapy. Together, our study uncovered a metabolic adaptation that fuels cancer resistance to nucleotide deficiency and positions the mTORC1 inhibitor, rapamycin, as a potential therapeutic strategy for transforming the management of chemotherapy-resistant lymphomas.

## Abstract 2

Resistant lymphomas recycle protein fragments to survive under chemotherapy, revealing new ways to overcome drug resistance.

## Introduction

Antimetabolites that target nucleotide biosynthesis, such as 6-mercaptopurine (6MP) and methotrexate, remain a cornerstone in chemotherapy treatment of acute lymphoblastic leukemia and B cell non-Hodgkin lymphoma (1–4). However, resistance to these therapies remains a major obstacle to curative outcomes (5). Understanding how cancer cells adapt to survive nucleotide deficiency is essential for developing new therapeutic strategies and overcoming drug resistance. 

Cancer cells monopolize nucleotide biosynthesis for their uncontrolled growth. Our previous work pinpointed the molecular mechanisms by which this was achieved downstream of Myc-driven lymphoma, which is implicated in more than 80% of Burkitt’s lymphoma cases (6). Specifically, Myc regulates the expression of phosphoribosyl pyrophosphate synthetase 2 (PRPS2), a rate-limiting enzyme in nucleotide biosynthesis. By generating what we believe is the first genetic loss-of-function mouse model for PRPS2 (*Prps2*^null^), we demonstrated that inhibition of PRPS2-dependent nucleotide production results in a profound block in *Myc*-driven tumorigenesis, while PRPS2 deficiency has no effects on normal development and physiology (7). However, despite the initial protection against lymphoma, approximately 40% of mice eventually develop tumors at later stages and become resistant to nucleotide deficiency (7). This model not only mirrors the therapeutic effects of antinucleotide drugs but also provides a powerful system to investigate the metabolic adaptations that underlie resistance to nucleotide-targeted therapies. Cancer cells are known to reprogram their metabolic networks to sustain growth under stress, including nutrient deprivation and chemotherapy-induced metabolic bottlenecks. While much attention has focused on how tumors adapt through changes in de novo nucleotide biosynthesis or salvage pathways (8), less is known about the metabolic rewiring that reprograms cellular signaling pathways to support survival under conditions of nucleotide deficiency.

In this study, employing an integrated approach combining metabolomics, RNA sequencing (RNA-seq), and ribosome profiling in the context of both genetically engineered mouse models (Eμ*-Myc*/^+^ *Prps2*^null^) and pharmacologically resistant human lymphoma cells, we uncovered a potentially novel dipeptide/mTOR complex 1 (mTORC1) signaling axis that enables lymphoma cells to bypass nucleotide stress. We found that resistance to nucleotide deficiency is associated with increased intracellular uptake of dipeptides enriched in essential amino acids, accompanied by an upregulation of the dipeptide transporter SLC15A3. Mechanistically, we found a selective interaction between SLC15A3 and mTOR proteins on the lysosome in resistant cancer cells, resulting in enhanced SLC15A3-dependent mTORC1 activity. We functionally show that genetically and pharmacologically derived resistant lymphomas, but not primary tumors, become sensitive to the mTORC1 inhibitor rapamycin, in culture and mouse models. In addition, we found that the vitamin D pathway acts as an upstream repressor of SLC15A3 transcription, and upregulation of SLC15A3 renders resistant cells vulnerable to active vitamin D3. Importantly, we observed an increase in SLC15A3 levels in lymphoma patient samples following antimetabolite chemotherapy. These findings uncover a crosstalk between dipeptide metabolism and the mTORC1 pathway that is exploited by cancer cells to adapt and grow under conditions of nucleotide deficiency. Most excitingly, we identify rapamycin, an already FDA-approved clinical compound, as a powerful new strategy to combat lymphoma resistance to antimetabolite chemotherapy, opening promising new avenues for targeted treatment.

## Results

### Nucleotide deficiency–resistant large B cell lymphoma upregulates dipeptide metabolism.

**Figure 1:**
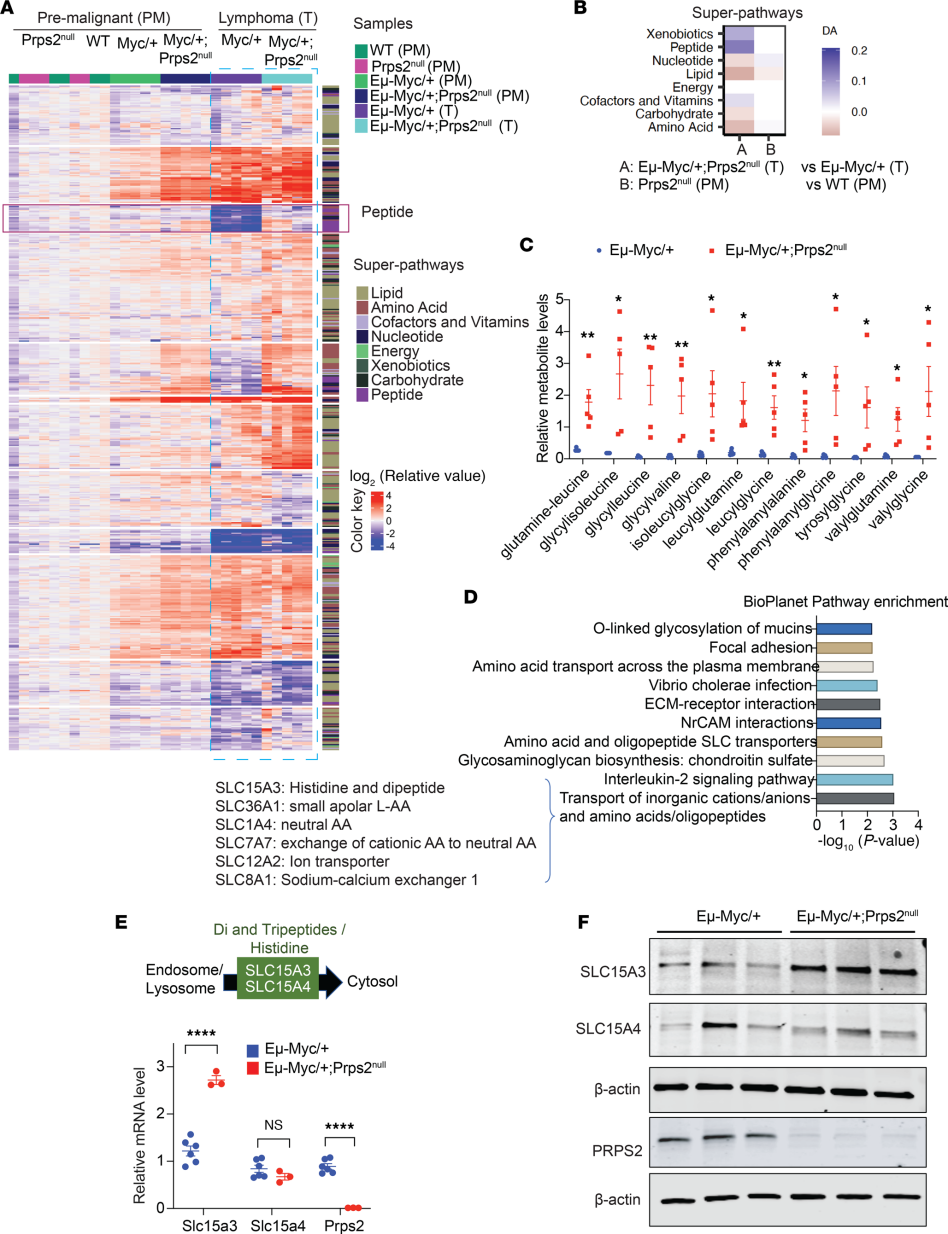
Genetically antinucleotide-resistant lymphoma specifically upregulates dipeptides and their transporter *SLC15A3*. (**A**) Heatmap showing relative metabolite levels (compared with WT B cells) of premalignant (PM) B cells from WT, *Prps2*^null^, Eμ*-Myc/^+^*, and Eμ*-Myc/^+^ Prps2*^null^ mice and lymphomas (T) from Eμ*-Myc/^+^*, and Eμ*-Myc/^+^ Prps2*^null^ mice (*n* = 5). (**B**) Differential abundance (DA) score for each superpathway (*n* = 5). A DA score approaching 1 indicates a coordinated increase in metabolite abundance within the pathway, whereas a score near –1 indicates widespread depletion. (**C**) Relative levels of significantly altered dipeptides between lymphoma from Eμ*-Myc/^+^ Prps2*^null^ and Eμ*-Myc/^+^* mice. (**D**) Top enriched pathways enriched in genes that are upregulated at both transcriptional and translational levels using BioPlanet pathway enrichment analysis. (**E**) Relative mRNA expression of indicated genes comparing lymphoma between Eμ*-Myc/^+^ Prps2*^null^ and Eμ*-Myc/^+^* mice. (**F**) Immunoblot analysis of indicated proteins in lymphoma from Eμ*-Myc/^+^ Prps2*^null^ and Eμ*-Myc/^+^* mice. β-Actin serves as the loading control. Individual data and mean ± SEM were presented in **C** and **E** and analyzed using 2-way ANOVA; *P < 0.05, **P < 0.01; ****P < 0.0001.

The *Myc* oncogene relies on the PRPS2-dependent nucleotide pathway for lymphoma initiation and progression. However, a percentage of *Myc*-transgenic mice eventually bypass nucleotide deficiency to form tumors (7). To investigate how large B cell lymphoma cancer cells rewire specific metabolic pathways to acquire resistance to PRPS2-mediated nucleotide deficiency, we performed an unbiased ultrahigh performance liquid chromatography-tandem mass spectrometry (UHPLC-MS) metabolomic profiling of B cells isolated from wild-type, *Prps2^null^*, Eμ*-Myc/^+^*, and Eμ*-Myc/^+^ Prps2*^null^ mice as premalignant samples, as well as lymphoma cells from Eμ*-Myc/^+^* and Eμ*-Myc/^+^ Prps2*^null^ mice as tumor samples. This profiling identified a total of 529 known metabolites, and their normalized levels (adjusted to Bradford protein concentration) across all mouse genetic backgrounds comparing premalignant cells and tumors, which are presented in the heatmap (Figure 1A and Supplemental Table 1; supplemental material available online with this article; https://doi.org/10.1172/JCI199709DS1). To provide functional context, metabolites were grouped into 8 superpathways based on broad biochemical domains: amino acid, nucleotide, lipid, peptide, carbohydrate, xenobiotic, energy, and cofactors/vitamins. Within tumors, a distinct cluster emerged that clearly distinguished Eμ-*Myc*/^+^ from Eμ-*Myc*/^+^ *Prps2*^null^ lymphomas and was enriched in metabolites belonging to the peptide superpathway (Figure 1A). To further quantify metabolic rewiring in response to *Prps2* deletion during *Myc*-driven tumorigenesis, we calculated the differential abundance (DA) score, a summary statistic commonly used in metabolomics enrichment analysis (Figure 1B). The DA score captures the overall direction of change within each pathway by comparing the number of significantly increased versus decreased metabolites. In Eμ-*Myc*/^+^ *Prps2*^null^ tumors, the DA score revealed an overall decrease in the nucleotide superpathway compared with Eμ-*Myc*/^+^ lymphomas (Figure 1B). Specifically, Eμ-*Myc*/^+^ *Prps2*^null^ tumors exhibited significant decreases in adenine, cytidine, and thymidine compared with Eμ-*Myc*/^+^ lymphomas, along with nonsignificant reductions in AICAR, inosine, and hypoxanthine, as well as in the nucleosides/bases adenosine, guanosine, and guanine (Supplemental Figure 1A and Supplemental Table 1). These data suggest that Myc-overexpressing tumors can circumvent the effects of nucleotide depletion independently of restoring nucleotide levels. Interestingly, the peptide superpathway exhibited the greatest increase among all superpathways in Eμ-*Myc*/^+^ *Prps2*^null^ tumors (Figure 1B). Particularly, we observed an augmentation in dipeptides — peptides composed of 2 amino acids (Supplemental Figure 1B). The significantly upregulated dipeptides predominantly contained glutamine or glycerol paired with essential amino acids, including leucine, isoleucine, and valine (Figure 1C). These findings suggest a specific metabolic reprogramming of dipeptide metabolism in nucleotide-deficient Eμ*-Myc/^+^ Prps2*^null^ lymphomas.

As Myc directly regulates both transcription and translation, to gain a deeper understanding of gene expression changes underlying the activation of specific metabolic pathways associated with resistance to nucleotide deficiency, we conducted RNA-seq and Ribo-seq analyses to profile the transcriptome and translatome of Eμ-*Myc*/^+^ *Prps2*^null^ lymphomas in comparison with Eμ-*Myc*/^+^ lymphomas. The analysis of these omics approaches revealed that the top enriched pathway in Eμ*-Myc/^+^ Prps2*^null^ lymphomas corresponds to Transport of amino acids/oligopeptides, which was upregulated at both the transcriptional and translational levels (Figure 1D, Supplemental Figure 1C, and Supplemental Table 2). This finding is consistent with our metabolomics results showing an increase in the peptide superpathway. Among the proteins belonging to this pathway, we identified SLC15A3, a transporter that is specific for dipeptides (9). B cells express 2 dipeptide transporters, SLC15A3 and SLC15A4 (10, 11). We further validated that only *Slc15a3*, but not *Slc15a4*, was upregulated in Eμ*-Myc/^+^ Prps2*^null^ lymphomas at the mRNA (Figure 1E) and protein levels (Figure 1F), while there was no increase of SLC15A3 or SLC15A4 in premalignant B cells (Supplemental Figure 1D). In addition to dipeptides, SLC15A3 also transports histidine (9). Consistent with increased *Slc15a3* expression, metabolites involved in histidine metabolism were also elevated in Eμ*-Myc/^+^ Prps2*^null^ lymphomas (Supplemental Figure 1E). Together, these findings demonstrate a distinct upregulation of dipeptide metabolism and its transporter, SLC15A3, in *Myc*-driven lymphoma upon nucleotide deprivation.

### Pharmacologically nucleotide-deficient B cell lymphomas upregulate SLC15A3 and dipeptide metabolism.

**Figure 2:**
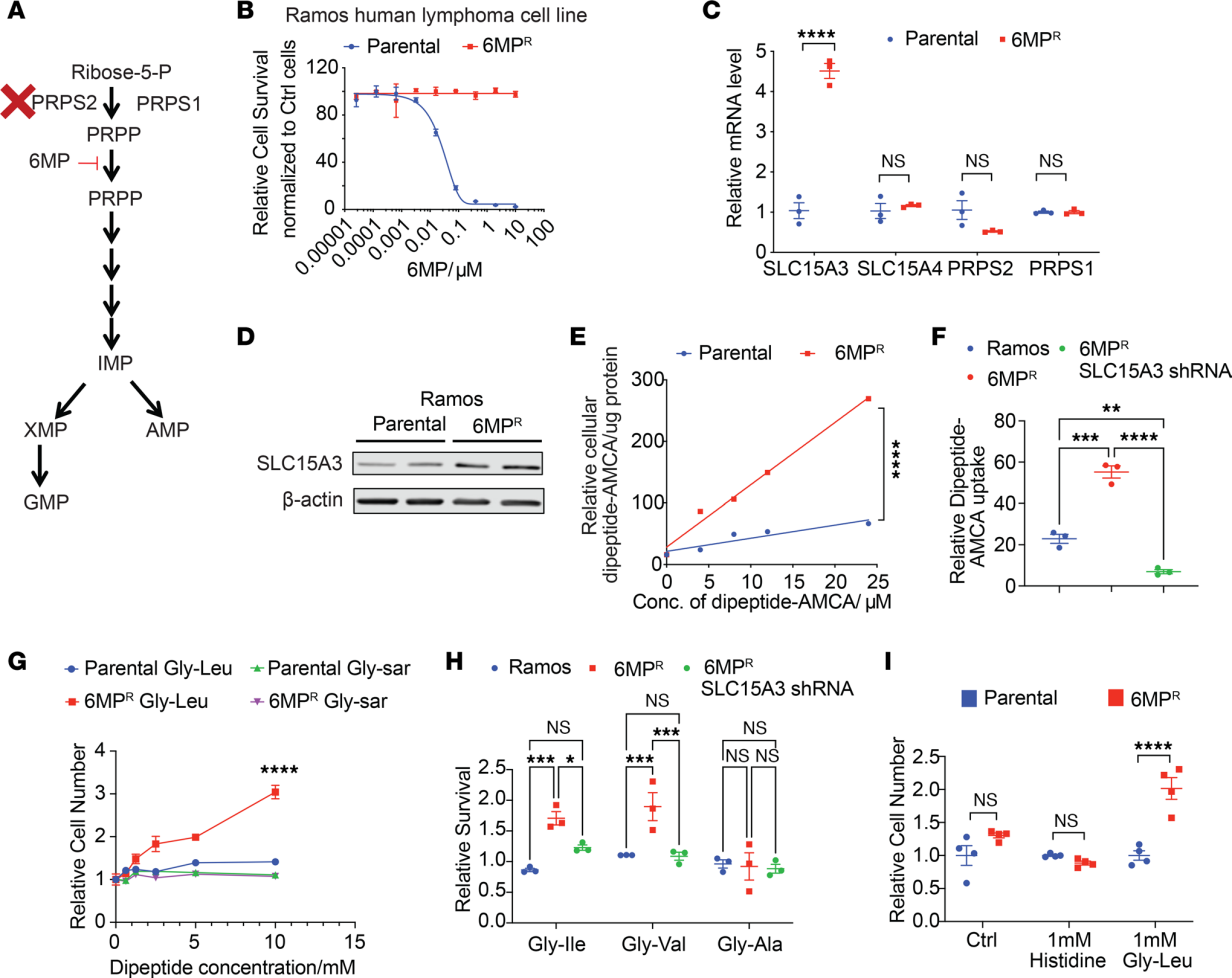
6MP-resistant human lymphomas upregulate SLC15A3 and dipeptide uptake. (**A**) Schematic of the de novo purine biosynthesis pathway starting from ribose-5-phosphate. (**B**) Relative cell survival of parental Ramos cells and 6MP-resistant (6MP^R^) Ramos cells treated with varying concentrations of 6MP for 2 days. (**C**) Relative mRNA expression levels of *SLC15A3* and *SLC15A4* in parental and 6MP^R^ Ramos cells. (**D**) Immunoblot analysis of SLC15A3 protein levels in parental and 6MP^R^ Ramos cells. β-Actin serves as the loading control. (**E**) Quantification of dipeptide-AMCA (7-amino-4-methylcoumarin-3-acetic acid, a fluorescent reference) levels in cell lysates from parental or 6MP^R^ Ramos cells treated with increasing concentrations of dipeptide-AMCA. (**F**) Relative dipeptide-AMCA in parental and 6MP^R^ Ramos cells or 6MP^R^ with SLC15A3 shRNA in basal condition. (**G**) Cell viability of parental and 6MP^R^ Ramos cells treated with varying concentrations of Gly-Leu or Gly-Sar in amino acid–free HBSS medium. (**H**) Cell viability of parental and 6MP^R^ Ramos cells treated with varying concentrations of 1 mM different dipeptides in amino acid–free HBSS medium. (**I**) Cell viability of parental and 6MP^R^ Ramos cells treated with 1 mM Gly-Leu or histidine in amino acid–free HBSS medium. Individual data and mean ± SEM were presented in **B**, **C**, and **E**–**I**, and **E**–**I** were analyzed using 2-way ANOVA; *P < 0.05, **P < 0.01; ***P < 0.001; ****P < 0.0001.

To strengthen the clinical significance of our data, we next sought to investigate whether the dipeptide pathway may also contribute to chemotherapy-induced resistance to nucleotide deficiency in human cancer cells. To this end, we generated an antimetabolite-resistant lymphoma cell line. We utilized Ramos cells, a human B lymphocyte cell line derived from a patient with Burkitt lymphoma commonly used as a model for B cell biology and Burkitt lymphoma. We treated Ramos cells with 6MP, a chemotherapy drug that targets the second step after PRPS2 within the purine biosynthesis pathway (Figure 2A) and is widely used in the treatment of lymphoma and leukemia (4, 12, 13). By subjecting the cells to a low-dose, long-term 6MP treatment followed by gradual dose escalation, we established a 6MP-resistant Ramos cell line (6MP^R^) capable of tolerating up to 10 μM 6MP (Figure 2B). Similar to Eμ*-Myc/^+^ Prps2*^null^ lymphomas, *SLC15A3* levels were significantly elevated in 6MP^R^ cells at both mRNA levels and protein levels (Figure 2, C and D), while *SLC15A4* remained unchanged. In addition, 6MP^R^ Ramos cells exhibited an increase of dipeptide uptake, as demonstrated by treatment with an AMCA-labeled dipeptide (Figure 2E and Supplemental Figure 2A). Knockdown of SLC15A3 in resistant cells significantly decreased the AMCA-labeled dipeptide uptake (Figure 2F and Supplemental Figure 2, B and C).

To investigate the functional role of upregulated dipeptides in 6MP-resistant cells, we assessed cell viability in amino acid–free HBSS supplemented with Gly-Leu, a dipeptide enriched in Eμ*-Myc/^+^ Prps2*^null^ lymphomas. Supplementation with Gly-Leu showed a dose-dependent effect on resistant cell survival but not with a nonhydrolysable dipeptide (Gly-Sar) (Figure 2G). We further examined the dipeptides with another 2 essential amino acids, Gly-Ile and Gly-Val, and found that all dipeptides with essential amino acids selectively enhanced the survival of 6MP^R^ cells but not parental cells. Importantly, downregulation of SLC15A3 attenuated this survival advantage (Figure 2H). In contrast, Gly-Ala, the dipeptide with a nonessential amino acid, failed to enhance resistant cells’ survival, as did supplementation with free histidine (Figure 2I). These findings suggest that antimetabolite-resistant lymphomas upregulate dipeptide metabolism and adopt a novel mechanism to utilize dipeptides as an alternative nutrient source to support survival.

### Elevated dipeptides enhance mTORC1 activation in resistant lymphoma through SLC15A3.

**Figure 3:**
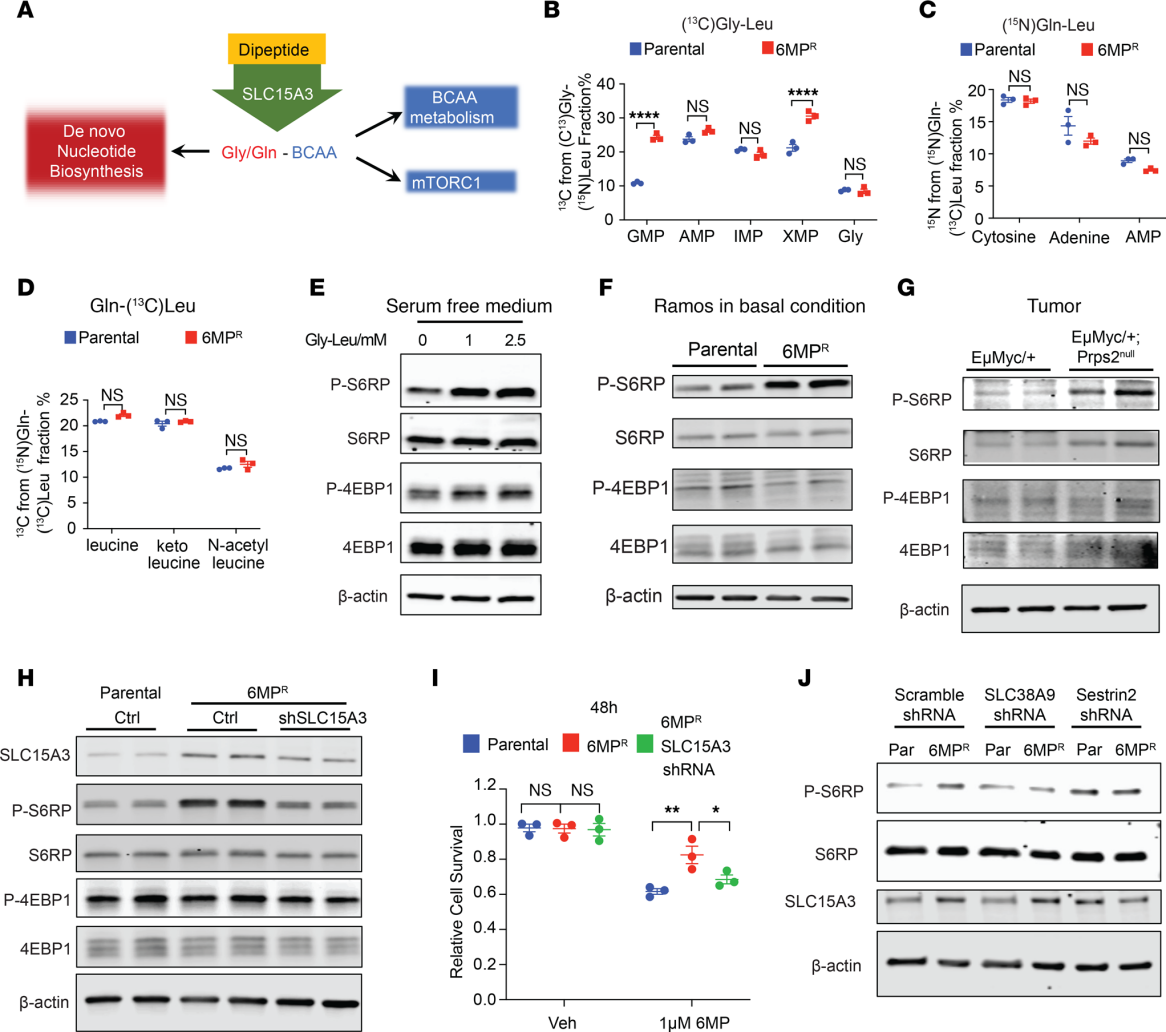
Elevated dipeptides are associated with enhanced mTORC1 activation in resistant lymphomas. (**A**) Schematic of pathways derived from dipeptide consisting of Gly/Gln with branched-chain amino acids (BCAA). (**B**) Percentage of ^13^C within different metabolites from parental or 6MP-resistant Ramos cells treated with (^13^C) Gly-Leu. (**C**) Percentage of ^15^N within different metabolites from parental or 6MP-resistant Ramos cells treated with (^15^N) Gln-Leu. (**D**) Percentage of ^13^C within different metabolites from parental or 6MP-resistant Ramos cells treated with Gln-(^13^C) Leu. (**E**) Immunoblot analysis of indicated proteins in Ramos cells treated with different concentrations of Gly-Leu in serum-free medium. (**F**) Immunoblot analysis of indicated proteins in parental or 6MP-resistant Ramos cells in full medium condition. (Internal control β-actin was the same one from Figure 2D.) (**G**) Immunoblot analysis of indicated proteins in Eμ-*Myc*/^+^ *Prps2*^null^ and Eμ-*Myc*/^+^ lymphomas. (**H**) Immunoblot analysis of the indicated proteins in parental and 6MP-resistant Ramos cells with or without shRNA-mediated knockdown of *SLC15A3* under basal conditions. (**I**) Relative cell survival of parental and 6MP-resistant Ramos cells with or without *SLC15A3* knockdown, treated with vehicle or 6MP for 2 days. (**J**) Immunoblot analysis of the indicated proteins in parental and 6MP-resistant Ramos cells with or without shRNA-mediated knockdown of *SLC38A9* or Sestrin2 under basal conditions. Individual data and mean ± SEM were presented in **B**–**D** and **I** and analyzed using 2-way ANOVA; *P < 0.05, **P < 0.01; ****P < 0.0001.

To investigate how dipeptides contribute to cell survival and resistance, we examined the utilization of dipeptides in antimetabolite-resistant lymphoma. We focused on the significantly upregulated dipeptides, which consist of glycine/glutamine and essential amino acids (Figure 1D). Glycine and glutamine are important for de novo nucleotide biosynthesis; essential amino acids such as leucine, isoleucine, and valine are substrates in branched-chain amino acid (BCAA) metabolism and potent activators of mTORC1 activation, a central regulator of cell proliferation and survival (Figure 3A). One hypothesis is that glycine and glutamine from dipeptides may contribute carbon (from glycine) and nitrogen (from glutamine) atoms, respectively, to fuel nucleotide biosynthesis. To test this, we performed isotope tracing experiments using (^13^C)-labeled glycine with leucine and (^15^N)-labeled glutamine with leucine. We detected significant increases in GMP and XMP levels in 6MP^R^ cells derived from the dipeptide of (^13^C)-labeled glycine with leucine but no changes in pyrimidines from the (AMIDE-^15^N)-glutamine–containing dipeptide (Figure 3, B and C). Next, we investigated whether the essential amino acid component of the dipeptides supports BCAA metabolism by tracing (^13^C)-labeled leucine with glutamine. However, no significant differences were found in BCAA pathway metabolites between parental and resistant cells (Figure 3D).

We then assessed the dipeptides’ effect on the mTORC1 signaling pathway. Similar to leucine alone, short-term Gly-Leu exposure in serum-free medium induced mTORC1 activity, as shown by increased phosphorylation of downstream targets S6 ribosomal protein (P-S6RP) and eukaryotic translation initiation factor 4E-binding protein 1 (P-4EBP1) (Figure 3E). In basal condition, 6MP^R^ cells exhibited elevated mTORC1 activity, as evidenced by a stronger phosphorylation of S6RP (Figure 3F), as well as its upstream kinase P-S6K and another mTORC1 target phospho-ULK1 (Ser757) (Supplemental Figure 3A). Similarly, genetically resistant Eμ*-Myc/^+^ Prps2*^null^ lymphoma displayed enhanced mTORC1 activation, particularly elevated P-S6RP levels and P-S6K levels (Figure 3G and Supplemental Figure 3A). As an increase in mTORC1 activity stimulates protein synthesis, we assessed protein synthesis in cells using a puromycin incorporation assay and found an increase in overall translation in 6MP^R^ cells (Supplemental Figure 3B). This result is also consistent with the large subset of genes that show increased translation (more than 2,500 genes with logTER > 0.5, adjusted *P* < 0.05 in ribosome profiling data) in Eμ*-Myc/^+^ Prps2*^null^ lymphomas (Supplemental Figure 3C and Supplemental Table 3). These genes are enriched in Translation and Ribosome proteins pathways, which are known to be activated downstream of mTOR. Further analysis of their 5′ untranslated regions (5′ UTRs) revealed significant enrichment of 5′ terminal oligopyrimidine (5′TOP) motifs, a hallmark of mTOR-dependent translational control (14) (Supplemental Table 4). Together, these results support an increase in mTOR pathway activity observed in resistant lymphoma.

To further investigate the role of dipeptide metabolism in bypassing nucleotide deficiency during *MYC*-driven lymphomagenesis, we used shRNA to downregulate SLC15A3 in 6MP^R^ Ramos cells. Excitingly, knockdown of *SLC15A3* back to parental levels led to a reduction in mTORC1 activity, specifically decreasing P-S6RP levels (Figure 3H), suggesting *SLC15A3* is required for the mTOR activation in the resistant cells. Additionally, *SLC15A3* downregulation reverted the resistance of 6MP^R^ cells to 6MP treatment (Figure 3I), indicating its essential role in mediating resistance adaptation. To determine whether SLC15A3-driven mTORC1 activation engages the canonical leucine-sensing pathway, we downregulated the lysosomal amino acid transporter SLC38A9 (15) and the leucine sensor Sestrin2 (16) in both parental and 6MPR Ramos cells (Supplemental Figure 3D). Under basal feeding conditions, SLC38A9 knockdown substantially reduced mTOR activity in resistant cells to levels comparable to parental cells, despite persistently elevated SLC15A3 (Figure 3J and Supplemental Figure 3E), indicating that enhanced mTOR activity in resistant cells is SLC38A9 dependent. In contrast, Sestrin2 downregulation increased mTOR activity in parental cells but had no further effect in resistant cells (Figure 3J and Supplemental Figure 3E), suggesting that SLC15A3 upregulation saturates the nutrient-induced mTOR pathway. These data support a model in which SLC15A3 promotes mTORC1 activation through canonical leucine-sensing mechanisms. Together, our findings suggest that lymphomas that bypass nucleotide deficiency reprogram their metabolism to increase dipeptide uptake and that dipeptides containing essential amino acids robustly activate the mTORC1 pathway in an SLC15A3-dependent manner to sustain growth under nucleotide-deficient stress.

### Enhanced SLC15A3 and mTOR colocalization in 6MP-resistant cells.

**Figure 4:**
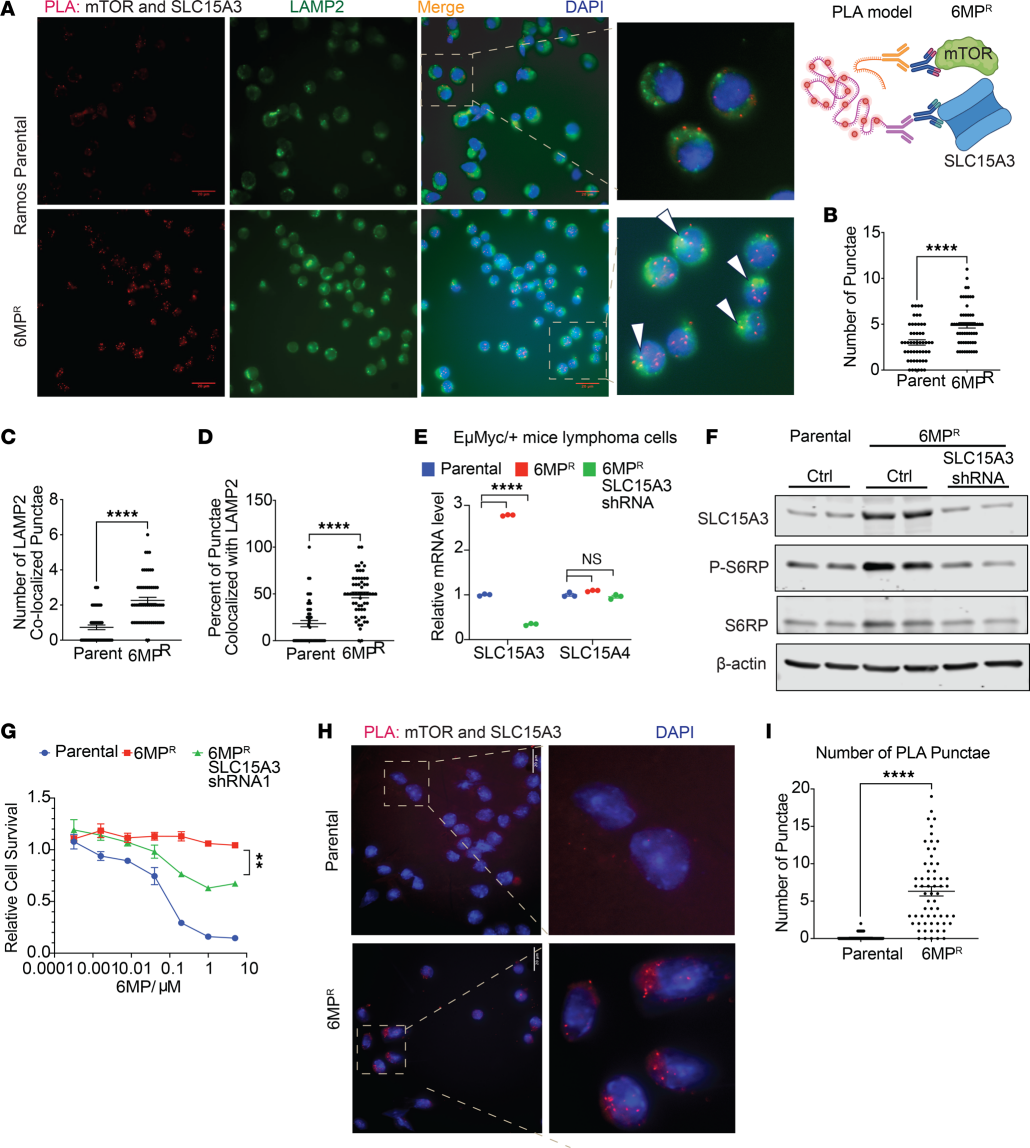
SLC15A3 colocalizes with mTOR in 6MP-resistant lymphoma. (**A**) Representative images and (**B**) quantification of proximity ligation assay (PLA) staining (red) using mTOR and SLC15A3 antibodies and LAMP2 (lysosomal-associated membrane protein 2) staining (green), which is a marker for lysosome, in parental 6MP-resistant Ramos cells; white arrows point at the colocalization. (**C**) Number of LAMP2 and PLA puncta colocalizations per cell in parental 6MP-resistant Ramos cells. (**D**) Percentage of puncta that colocalize with LAMP2 in parental 6MP-resistant Ramos cells. (**E**) Relative mRNA levels of *Slc15a3* and *Slc15a4* in parental and 6MP-resistant Eμ-*Myc/^+^* lymphoma cells without or with shRNA-mediated knockdown of *Slc15a3*. (**F**) Immunoblot analysis of the indicated proteins in parental and 6MP-resistant Eμ-*Myc/^+^* lymphoma cells under basal conditions. (**G**) Relative cell survival of parental and 6MP-resistant Eμ-*Myc/^+^* lymphoma cells with or without shRNA-mediated *Slc15a3* knockdown, treated with vehicle or 6MP for 2 days. (**H**) Representative images and (**I**) quantification of PLA staining (red) using mTOR and SLC15A3 antibodies and LAMP2 staining (green) in parental 6MP-resistant Eμ-*Myc/^+^* lymphoma cells. Individual data and mean ± SEM were shown in **B**–**E**, **G**, and **I**. **B**, **C**, and **I** were analyzed using 2-sided *t* test, and **E** and **G** were analyzed using 2-way ANOVA; **P < 0.01; ****P < 0.0001.

SLC15A3 is known to localize to late endosomes and lysosomes, where it facilitates the export of dipeptides/histidine out from the lysosome to the cytosol (9, 17). mTORC1 is activated at the lysosomal surface in response to amino acid sensing, a process mediated by direct interactions with lysosomal amino acid transporters such as SLC38A9, which exports essential amino acids such as leucine from the lysosome (18). We hypothesized that SLC15A3 may regulate mTORC1 activation by interacting with mTOR at the lysosome and transporting dipeptides containing essential amino acids. To test this hypothesis, we used a proximity ligation assay (PLA), a molecular technique that detects protein-protein interactions occurring within a few nanometers. Strikingly, while interactions between SLC15A3 and mTOR were undetectable in parental cells, we observed a robust PLA signal, visualized as distinct red puncta, in 6MP^R^ cells (Figure 4, A and B, and Supplemental Figure 4, A and B). Co-immunoprecipitation confirmed the colocalization between SLC15A3 and mTOR, particularly in 6MP^R^ cells, as confirmed by quantification (Supplemental Figure 4, C and D). Importantly, these PLA puncta partially colocalized with the lysosomal marker LAMP2 (Figure 4, A, C, and D), and this increased in the resistant cells, revealing that SLC15A3 and mTOR are colocalized on the lysosome. Furthermore, we examined whether SLC15A3 lysosomal localization is regulated by nutrient availability. PLA analysis between SLC15A3 and LAMP2 revealed increased lysosomal localization of SLC15A3 following serum and amino acid starvation, as indicated by the presence of SLC15A3–LAMP2 puncta. In contrast, serum refeeding abolished this colocalization (Supplemental Figure 4, E and F), which is consistent with the lower puncta of SLC15A3 and mTOR we observed in the basal parental cells. These findings suggest that SLC15A3 localization and its interaction with mTOR are steps of an adaptive response to stress, such as nutrient or nucleotide deprivation.

We extended this analysis to a transplantable Eμ*-Myc/^+^* lymphoma model derived from Eμ*-Myc/^+^* mice (19), and using the same method, we generated a 6MP^R^ line (Figure 4G). These resistant lymphomas recapitulated key features observed in Ramos cells, including increased SLC15A3 but not SLC15A4 expression (Figure 4E), enhanced mTOR pathway as evidenced by elevated P-S6RP levels, and reduced mTORC1 activity upon knockdown of SLC15A3 and restored sensitivity to 6MP (Figure 4, F and G). Consistently, SLC15A3 showed increased colocalization with mTOR in these resistant Eμ*-Myc/^+^* lymphoma cells (Figure 4, H and I). In addition, we assessed lysosomal acidification and function using acridine orange staining, which revealed increased lysosomal acidification in both resistant Ramos cells and the resistant Eμ*-Myc/^+^* lymphoma line (Supplemental Figure 5, A and B). These findings support a model in which antimetabolite-resistant lymphoma cells cope with stress by upregulating SLC15A3, thereby driving mTORC1 activation through direct engagement of mTOR at the lysosome.

### Antimetabolite-resistant lymphoma is sensitive to rapamycin.

**Figure 5:**
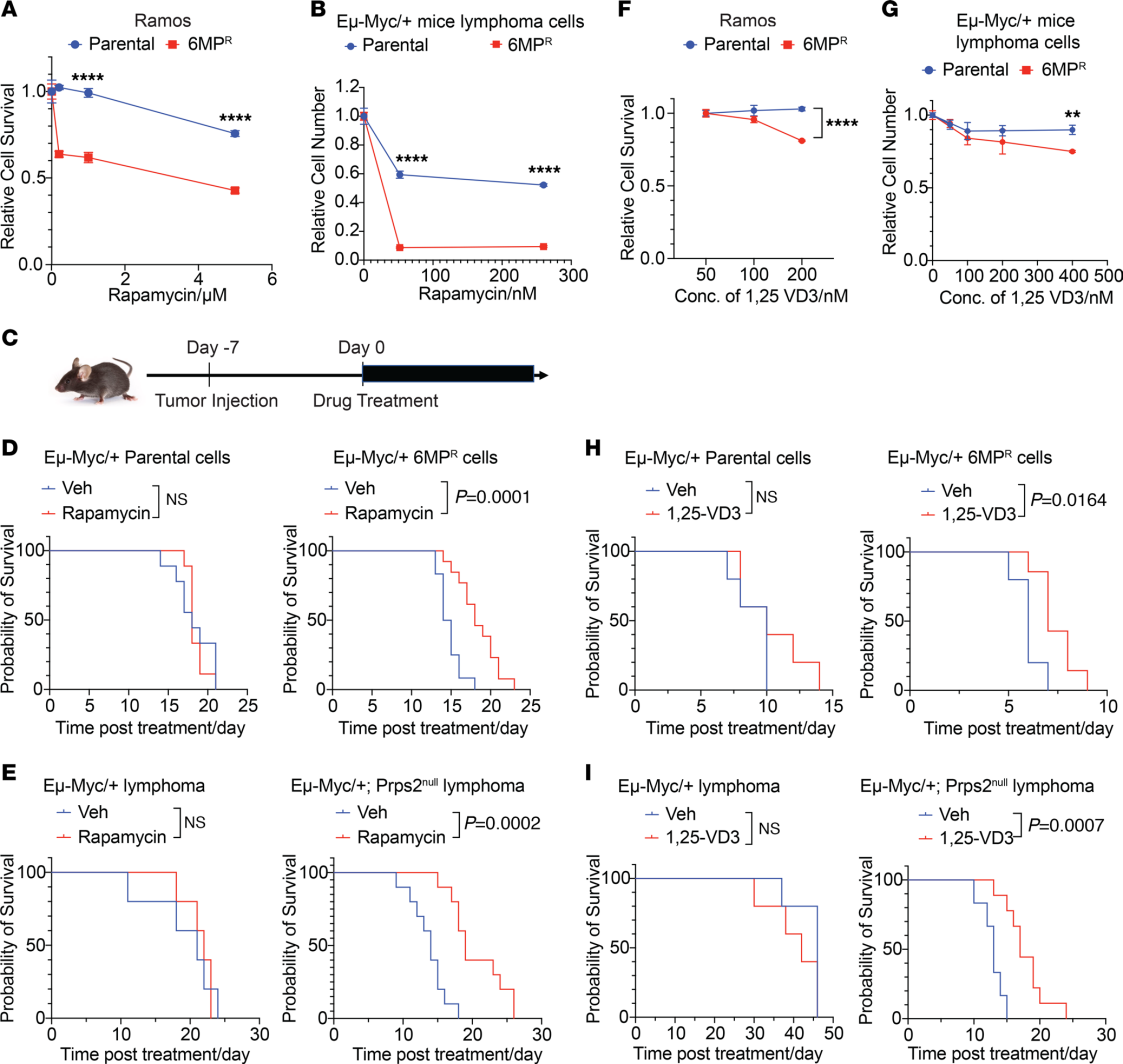
Resistant lymphomas are more sensitive to rapamycin and active VD3. (**A**) Relative cell survival of parental and 6MP-resistant Ramos cells and (**B**) Eμ-*Myc/^+^* lymphoma cells treated with different concentrations of rapamycin for 2 days. (**C**) Scheme of allograft lymphoma mouse model with the drug treatment. (**D**) Kaplan-Meier survival curves of wild-type B6 mouse tail vein injected with parental (*n* = 5 for vehicle [veh], *n* = 9 for drug) or 6MP-resistant Eμ-*Myc/^+^* lymphoma (*n* = 12 for veh, *n* = 6 for drug) and (**E**) wild-type B6 mouse tail vein injected with Eμ-*Myc*/^+^ (*n* = 5 for veh, *n* = 5 for drug) or Eμ-*Myc*/^+^ *Prps2*^null^ lymphoma (*n* = 10 for veh, *n* = 10 for drug), treated with vehicle or rapamycin starting 1 week postinjection. (**F**) Relative cell survival of parental and 6MP-resistant Ramos cells and (**G**) Eμ-*Myc/^+^* lymphoma cells treated with different concentrations of 1,25-VD3 for 2 days. (**H**) Kaplan-Meier survival curves of wild-type B6 mouse tail vein injected with parental (*n* = 5 for veh, *n* = 5 for drug) or 6MP-resistant Eμ-*Myc/^+^* lymphoma (*n* = 5 for veh, *n* = 6 for drug) and (**I**) wild-type B6 mouse tail vein injected with Eμ-*Myc*/^+^ (*n* = 5 for veh, *n* = 5 for drug) or Eμ-*Myc*/^+^ *Prps2*^null^ lymphoma (*n* = 6 for veh, *n* = 9 for drug), treated with vehicle or 1,25-VD3 starting 1 week postinjection. Individual data and mean ± SEM were shown in **A**, **B**, **F**, and **G** and analyzed using 2-way ANOVA; **D**, **E**, **H**, and **I** were analyzed using log-rank (Mantel-Cox) test; **P < 0.01; ****P < 0.0001. 1,25-VD3, 1,25-dihydroxyvitamin D3.

Resistant lymphomas exhibited elevated P-S6RP, leading us to hypothesize that they might be sensitive to the mTOR inhibitor, rapamycin, which primarily targets the S6K/P-S6RP axis but not P-4EBP/eIF4E axis of the mTORC1 pathway (20–23). To test this hypothesis, we treated cells with a titration of rapamycin. While rapamycin had little effect on parental Ramos cells, it significantly impaired the survival of 6MP^R^ cells (Figure 5A). In contrast, consistent with previous studies (24), ATP-competitive mTOR inhibitors that suppress SK6/P-S6RP and the 4EBP/eIF4E axis, such as Rapalink, reduced viability in both parental and resistant cells, with resistant cells showing greater sensitivity (Supplemental Figure 6A). Similarly, 6MP^R^ Eμ-*Myc*/^+^ lymphomas also displayed marked sensitivity to rapamycin (Figure 5B) but similar high sensitivity to Rapalink (Supplemental Figure 6B). These findings demonstrate that SLC15A3-driven mTORC1 activation not only promotes resistance to 6MP but also creates a therapeutic vulnerability to rapamycin.

To assess therapeutic responses of rapamycin in vivo, we established an allograft lymphoma model by intravenously injecting 6MP^R^ Eμ*-Myc/^+^* cells into wild-type B6 mice (Figure 5C). While rapamycin treatment had little effect on mice bearing parental Eμ*-Myc/^+^* lymphoma, it significantly prolonged the survival of mice harboring 6MP^R^ tumors (Figure 5D). We next tested the genetically nucleotide deficiency–resistant lymphoma model by injecting Eμ*-Myc/^+^* or Eμ*-Myc/^+^ Prps2*^null^ lymphoma into the wild-type B6 mice. Consistent with the pharmacologically resistant model, rapamycin selectively extended the lifespan of mice bearing the Eμ*-Myc/^+^ Prps2*^null^ lymphoma (Figure 5E), which showed no effect on Eμ*-Myc/^+^* tumors. These data support the effectiveness of rapamycin in targeting lymphoma under conditions of nucleotide deficiency.

### Antimetabolite-resistant lymphoma is sensitive to active vitamin D3.

To explore potential upstream regulators of SLC15A3 in resistant cells for therapeutic tractability, we sought to identify transcription regulators of SLC15A3 by interrogating multiple publicly available transcription factor (TF) datasets, including ARCHS4 TF Coexpression, JASPAR PWM Human 2025, and TF Perturbation–Expression. Across these independent analyses, the vitamin D receptor (VDR) emerged as a consistently predicted TF associated with SLC15A3 expression (Supplemental Table 5 and Supplemental Figure 6C). VDR is a nuclear receptor that functions as a ligand-activated TF upon binding active VD3 (such as 1,25-VD3, a strong VDR ligand), and binds vitamin D response elements in the promoter region to modulate transcription of target genes. We found several strong vitamin D response elements within the SLC15A3 promoter region (Supplemental Figure 6D). VDR has been shown to act as either an activator or repressor depending on the target genes and cell type (25, 26). Interestingly, in our metabolomics data, we found that the VDR ligand 1,25-VD3 was significantly reduced in both 6MP-resistant cells and Eμ-*Myc*/^+^ *Prps2*^null^ tumors (Supplemental Figure 6, E and F). Given that SLC15A3 was upregulated in these resistant cells, we hypothesized that, in this context, VDR may act as a repressor and that reduced levels of its ligand result in increased SLC15A3 expression.

To test this hypothesis, we treated cells with 1,25-VD3 and found that activating the VD3 pathway significantly reduced SLC15A3 expression, with a more pronounced effect in resistant cells, but showed no effect on SLC15A4 (Supplemental Figure 6G). Functionally, 1,25-VD3 treatment attenuated the mTOR pathway specifically in resistant cells (Supplemental Figure 6H) and increased cell death in these cells (Figure 5, F and G). Overexpression of SLC15A3 cDNA rescued this sensitivity (Supplemental Figure 6, I and J), while partial downregulation of SLC15A3 increased sensitivity to 1,25-VD3 (Supplemental Figure 6K). Excitingly, in allograft lymphoma models, active VD3 treatment selectively prolonged the lifespan of mice bearing 6MP^R^ Eμ*-Myc/^+^* cells or Eμ*-Myc/^+^ Prps2*^null^ lymphoma but not parental lymphoma (Figure 5, H and I). Together, these observations suggest a role for VDR signaling in modulating SLC15A3 expression and function in resistant lymphoma.

### SLC15A3 levels positively correlate with antimetabolite treatment in lymphoma patients and various human cancers.

**Figure 6:**
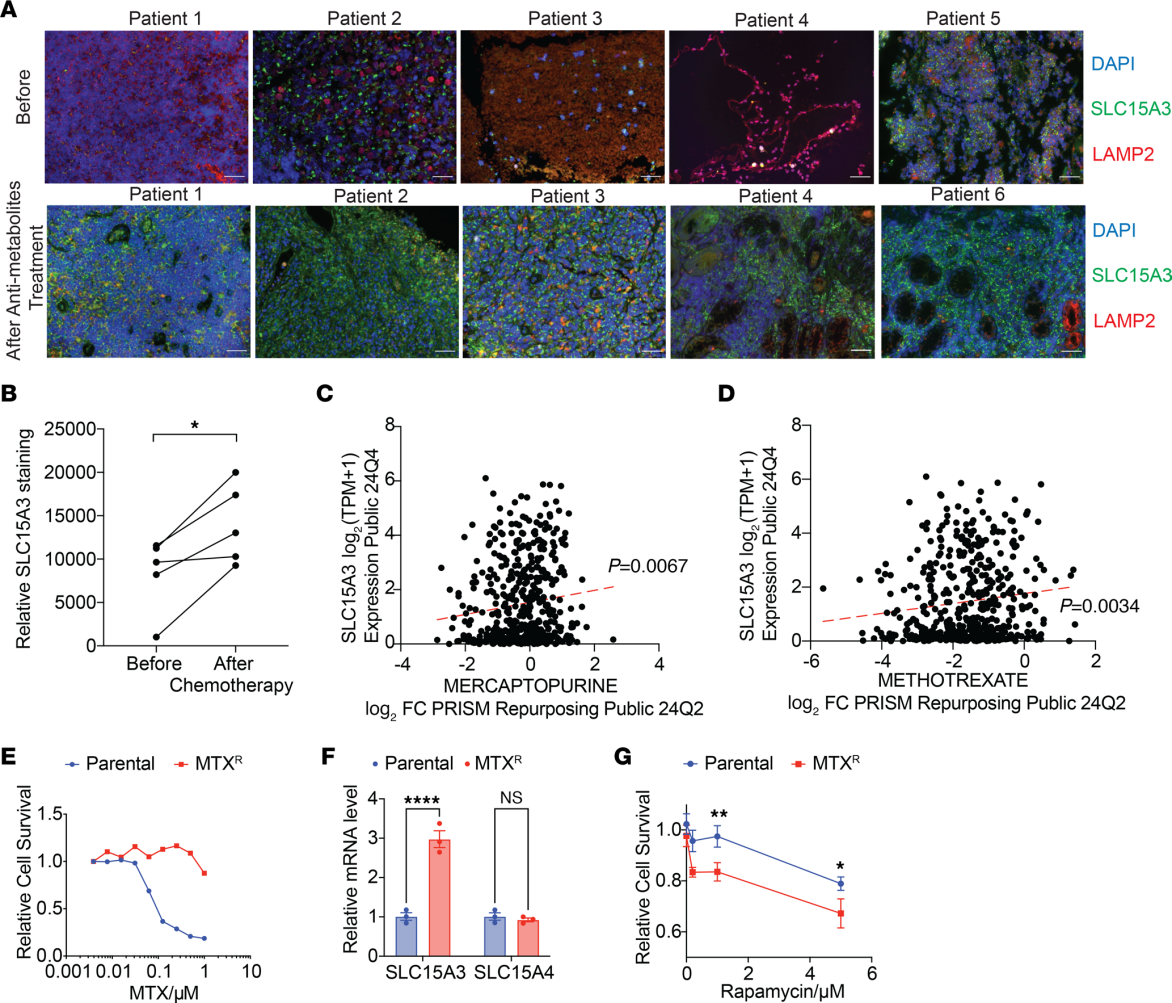
Targeting nucleotide biosynthesis upregulates SLC15A3 in lymphoma patients’ biopsies and various human cancers. (**A**) Representative image of immunofluorescence of SLC15A3 in lymphoma patients’ biopsies collected before and after antimetabolite chemotherapy. Scale bar = 100 µm. (**B**) Quantification of SLC15A3 levels in lymphoma patients’ biopsies collected before and after antimetabolite chemotherapy. (**C**) Two-dimensional plot of the log_2_ *SLC15A3* expression levels compared with the sensitivity of mercaptopurine (6MP) in 487 human cell lines based on PRISM Repurposing Screen. (**D**) Two-dimensional plot of the log_2_ *SLC15A3* expression levels compared with the sensitivity of methotrexate in 491 human cell lines based on PRISM Repurposing Screen. (**E**) Relative cell survival of parental Ramos cells and methotrexate-resistant (MTX^R^) Ramos cells treated with varying concentrations of methotrexate for 2 days. (**F**) Relative mRNA expression levels of *SLC15A3* and *SLC15A4* in parental and methotrexate-resistant Ramos cells. (**G**) Relative cell survival of parental and methotrexate-resistant Ramos cells treated with different concentration of rapamycin for 2 days. Individual data and mean ± SEM were shown in **B**–**G**. **B** was analyzed using 2-sided *t* test, **C** and **D** were analyzed using simple linear regression, and **F** and **G** were analyzed using 2-way ANOVA; **P < 0.01; ****P < 0.0001.

To evaluate the clinical relevance of these findings, we analyzed patient lymphoma biopsies collected before and after antimetabolite chemotherapy. Strikingly, immunofluorescence analysis showed a significant increase of SLC15A3 expression in posttreatment samples from the same patients (Figure 6, A and B), suggesting a similar increase of SLC15A3-dipeptide metabolism occurs in lymphoma after antimetabolite therapy. To extend these observations across different cancer types following antimetabolite treatment, we examined nearly 500 human cancer cell lines using the PRISM Repurposing Public 24Q2 screen. SLC15A3 transcript expression positively correlated with resistance to 6MP (Figure 6C and Supplemental Table 6). A similar significant correlation was observed with methotrexate, another widely used antimetabolite (Figure 6D and Supplemental Table 7). We further generated a methotrexate-resistant Ramos cell line (MTXR; Figure 6E) capable of tolerating up to 1 μM methotrexate. Consistent with 6MP^R^ data, MTXR cells showed increased SLC15A3, but not SLC15A4, expression (Figure 6F) and showed enhanced sensitivity to rapamycin (Figure 6G). These results indicate that antimetabolite-resistant cancer cells upregulate the dipeptide-SLC15A3/mTOR axis and highlight rapamycin, an FDA-approved mTOR inhibitor, as a potential therapeutic strategy for patients with antimetabolite-resistant lymphoma.

## Discussion

Resistance to antimetabolite chemotherapies, such as 6MP and methotrexate, remains a significant clinical challenge in the treatment of B cell lymphomas (1, 5). These drugs target nucleotide biosynthesis, exploiting the dependency of rapidly dividing cells on nucleotide availability. While resistance is often attributed to restoration of nucleotide pools via de novo or salvage pathways, our study uncovers a distinct metabolic adaptation: resistant lymphoma cells increase dipeptide uptake through the peptide transporter SLC15A3, which reactivates mTORC1 signaling to boost tumor proliferation under nucleotide stress.

Dipeptides have been repeatedly detected in cancer metabolomic profiles, and elevations of specific dipeptides have been reported across tumor types (27–29); however, their biological functions remain unclear. Our study reveals a functional role for dipeptides containing essential amino acids in regulating the mTOR pathway. This reframes the notion that these metabolites are simply passive by-products of cancer growth. They are instead serving as active modulators of cancer cell metabolism.

Mechanistically, we show that the specific peptide transporter SLC15A3 links the dipeptide and mTORC1 pathway through its direct interaction with mTOR on the lysosome. mTORC1 activation has previously been attributed to amino acid transporters such as SLC38A9 and SLC36A1 (18, 30). Our findings expand this paradigm by showing that SLC15A3 physically interacts with mTOR and promotes its activation in an SLC38A9-dependent manner. Notably, a related transporter, SLC15A4, has also been shown to interact with mTOR in leukemia (31), suggesting that peptide transporters represent an underexplored class of mTOR regulators in hematologic malignancies.

Previous studies have established a positive correlation between the mTOR pathway and purine biosynthesis (32, 33); intriguingly, our results uncover a recovery feedback mechanism in which mTOR activation drives the resistance to purine deprivation by enhancing the S6K/P-S6RP downstream pathway. Our previous studies showed that mTOR-dependent P-4EBP1 is highly activated and promotes the survival of *Myc*-driven lymphoma, rendering these cells largely insensitive to rapalog, which does not effectively inhibit the 4EBP1 arm of mTORC1 signaling (24). Our current model suggests that resistant cells preferentially enhance the S6K/P-S6RP axis of mTORC1 signaling through dipeptide-SLC15A3 to sustain survival under nucleotide deficiency stress. Importantly, this adaptive rewiring confers a potentially previously unrecognized vulnerability to rapamycin, which primarily targets the S6K/P-S6RP axis, in nucleotide deficiency–resistant lymphoma. Rapamycin is FDA approved for transplant rejection and lymphangioleiomyomatosis, but its use in cancer has been limited because of modest clinical efficacy, especially when used as monotherapy (22, 34). Consistent with these prior findings, our study shows that rapamycin alone does not significantly extend survival in mice bearing lymphoma allografts. However, in 6MP-resistant lymphoma models or *Prps2*-deficient tumors, rapamycin effectively impairs tumor growth, highlighting a potential new use in antimetabolite-resistant lymphomas. Moreover, our findings suggest that SLC15A3 could serve as a biomarker for rapamycin sensitivity.

Our study identified that the vitamin D3 pathway is one of the upstream regulators of SLC15A3 that represses its transcription in *Myc*-driven lymphoma. Importantly, resistant cells exhibited a deficiency in active vitamin D3 and showed increased sensitivity to active vitamin D3, rendering a new therapeutic vulnerability. In recent years, vitamin D deficiency has been reported as a negative prognostic factor in aggressive B cell lymphomas and is strongly associated with inferior outcomes, including increased risk of disease progression, higher mortality, and reduced efficacy of chemotherapy (35–37). Our study suggests that SLC15A3, the downstream target of VDR, may provide a mechanistic link between vitamin D deficiency and poor clinical outcomes. Indeed, VDR has been shown to act as a tumor repressor partially by inhibiting mTOR activity (38, 39), consistent with our VDR/SLC15A3/mTOR pathway model in regulating resistant lymphoma survival.

Together, our work identifies a potentially previously unrecognized metabolic mechanism driving resistance to antimetabolite therapy and reveals SLC15A3 as both a functional driver and a potential biomarker of rapamycin sensitivity in B cell lymphoma.

## Methods

### Sex as a biological variable.

For clinical samples and animal models, both sexes were involved. The findings were expected to be relevant to both sexes.

### Materials.

We used 1a,25-Dihydroxyvitamin D3 ≥99% (HPLC) (Sigma D1530), Leu-Gly (Sigma L9625), Gly-Sar (Sigma G3127-1G), Gly-Ile (Avantor G-2135), Gly-Val (Avantor G-2250), l-histidine (Sigma 97062-598), and rapamycin (Thermo Fisher Scientific 502028702).

### Mice.

C57BL/6 Eμ*-Myc/^+^* mice have been previously characterized (40), and *Prprs2^null^* mice have been previously characterized (7). To generate Eμ*-Myc/^+^ PRPS2*^null^ mice, we intercrossed Eμ*-Myc/^+^* mice to *Prprs2*^null^ mice (7). Mouse cohorts consisted of littermates or similar litters weaned together. Eμ*-Myc/^+^* clonal B cell line collection was described before (41). Briefly, Eμ*-Myc/^+^* or Eμ*-Myc/^+^ PRPS2*^null^ mice bearing lymphoma were euthanized, and lymph nodes were immediately collected on ice. Tissues were minced and passed through a 40 μm cell strainer to obtain single-cell suspensions. Cells were resuspended in cold ACK erythrocyte lysis buffer (Thermo Fisher Scientific, A1049201) for 1 minute, then centrifuged at 300*g* for 5 minutes and washed once with PBS. Cells were either frozen in cryopreservation medium and stored in liquid nitrogen for future injection or analysis or used immediately for downstream experiments.

### Genotyping protocol for Prprs2^null^ mice.

Eμ*-Myc/^+^ Prprs2*^null^ mice were genotyped using Forward 5′-ACATTGCCATAAGGAATTATCAGAG-3′ and Reverse 5′- GGCGCCAGCCTGCTTT-3′ to detect the mutant allele and Forward 5′-TGCCAGTTATCACCGCTCA-3′ and Reverse 5′-GCTGCCCACACTTCACTCTT-3′ to detect the wild-type allele.

### Allograft lymphoma mouse models.

To generate mouse models, cryopreserved stocks of Eμ*-Myc/^+^* or Eμ*-Myc/^+^ Prprs2*^null^ lymphoma cells were thawed and washed once in PBS. Cells were counted and resuspended in Dulbecco’s PBS (DPBS) for tail vein injection. A total of 500,000 live cells were injected intravenously into 8-week-old male C57BL/6 mice purchased from The Jackson Laboratory. For experiments using cultured Eμ*-Myc/^+^* or 6MP-resistant Eμ*-Myc/^+^* lymphoma cells, cells were washed once with PBS, then resuspended in DPBS; 0.1 million live cells were injected intravenously into 8-week-old male C57BL/6 mice. Mice were monitored for lymphoma development by palpation every other day. Once the lymphoma tumors became palpable (approximately 1 or 2 weeks after tumor cell injection), mice (5 per group) were dosed by intraperitoneal injection with 4 mg/kg rapamycin (Selleckchem S1039) or vehicle 3 times per week (Monday, Wednesday, and Friday) until the survival endpoint or treated with 50 pg/g 1,25-VD3 (Sigma 679101) 3 times per week (Monday, Wednesday, and Friday). Rapamycin was obtained from Selleckchem, prepared as a 25× solution in DMSO, and stored at –20°C in aliquots along with aliquots of DMSO until use. For injection, aliquots were thawed and diluted in a vehicle of 5% Tween 80 and 5% polyethylene glycol in 0.9% NaCl to yield a final concentration of 4% DMSO. The maximum allowable tumor size (2 cm in diameter) was not exceeded in any study. No animals or data points were excluded from the analyses.

### B cell and lymphoma isolation.

Mouse B cells and B lymphocytes were isolated from single-cell spleen or lymph node suspensions using the EasySep Mouse T Cell Isolation Kit (STEMCELL Technologies, 19851) and EasySep Mouse B Cell Isolation Kit (STEMCELL Technologies, 19854), respectively, according to the manufacturer’s instructions. Briefly, spleen was mechanically dissociated in PBS containing 2% FBS, passed through a 70 μm strainer, pelleted at 300*g* for 10 minutes, and resuspended at 1 × 10^8^ nucleated cells/mL in PBS + 2% FBS + 1 mM EDTA. Normal rat serum (50 μL/mL) was added, followed by the isolation cocktail (50 μL/mL) and incubation for 10 minutes at room temperature. Streptavidin RapidSpheres were vortexed and added at 75 μL/mL with incubation for 2.5 minutes at room temperature. Then samples were brought to the recommended volume with buffer and placed in an EasySep magnet for 2.5 minutes. The enriched (unlabeled) T cell or B cell fraction was collected by pouring the supernatant into a new tube and used immediately for downstream assays.

### Co-immunoprecipitation assays.

For co-immunoprecipitation, we used protein G beads and an mTOR (Cell Signaling 2972S) antibody for pull-down. Following gel electrophoresis, proteins were transferred onto a nitrocellulose membrane. The membrane was blocked in TBST buffer (pH 7.5; 0.02 M Tris-HCl, 0.15 M NaCl, and 0.05% Tween 20) containing albumin (3%), blotted with a primary antibody (overnight at 4°C), washed in TBST, and incubated for 30 minutes with a secondary antibody conjugated to HRP. mTOR (Cell Signaling 2972S) and SLC15A3 (Proteintech 20866) antibodies were used for immunoblotting.

### Mass spectrum metabolomics analysis.

The metabolites from B cells and lymphoma tumors were measured with the Metabolon analytical system. A nontargeted semiquantitative liquid chromatography–tandem mass spectrometry (LC-MS/MS) platform was applied for the identification of structurally named and unknown molecules (42). All normalized relative ion counts were log-transformed, and the remaining data were imputed with the minimum value on a per-metabolite basis and reported in relative units.

To assess pathway-level shifts in metabolite abundance, we calculated a DA score for each (super)pathway (43). Specifically, DA analysis was first performed for each comparison group using MetaboAnalyst (version 6.0) (44). Metabolites with an absolute log_2_ fold-change greater than 1 were considered differentially abundant and included in subsequent DA score analysis. For each pathway in each comparison group, the DA score was calculated as: DA = (number of increased metabolites – number of decreased metabolites)/total number of measured metabolites in the pathway. A DA score approaching 1 indicates a coordinated increase in metabolite abundance within the pathway, whereas a score near –1 indicates widespread depletion.

### 1,25-VD3 measurement.

For the ELISA, the cells or tumors were processed according to the manufacturer’s instructions of the Universal 1,25-dihydroxyvitamin D3(DVD/DHVD3) ELISA Kit (NBP2-82432, NOVUS Biologicals), and protein concentration were measured using BCA assay (Thermo Fisher Scientific).

### Immunoblot analysis.

Immunoblot analysis was performed on samples lysed in RIPA lysis buffer (EMD Millipore Corp, no. 20-188) with the addition of PhosSTOP (Roche, no. 4906837001) and Complete Mini proteasome inhibitors (Roche, no. 4693124001) using standard procedures. Briefly, lysates were iced and vortexed for 30 minutes, then centrifuged, and an aliquot of supernatant was used for BCA assay to determine protein concentration. Western blot analysis was performed using commercial antibodies at a 1:1,000 ratio for SLC15A3 (Proteintech no. 20866), β-actin (Sigma, no. A5316), P-S6RP (Cell Signaling, no. 2215S), S6RP (Cell Signaling, no. 2317), mTOR (Cell Signaling, no. 2972), 4EBP1 (Cell Signaling, no. 9644), P-4EBP1 (Cell Signaling, no. 2855), P-S6K (Cell Signaling no. 9234S), S6K (Cell Signaling no. 9202), P-ULK1 (Cell Signaling no. 6888S), ULK1 (Invitrogen no. MA557310), puromycin (Kerafast no. EQ0001), GAPDH (Cell Signaling no. 2118S), PRPS2 (Genetex no. GTX104048), and PRPS1 (Abcam no. ab137577). Secondary antibodies used were goat anti-Mouse (Li-Cor, no. 926-68070) and goat anti-rabbit (Li-Cor, no. 926-32211). Where mentioned, protein abundance was determined using ImageJ software (NIH) to analyze the optical density of Western blots normalized to loading control.

### Case selection.

Database search was performed at UCSF for cases of non-Hodgkin B cell lymphoma between 2003 and 2024. This search identified 5 patients with pathologically confirmed non-Hodgkin B cell lymphoma with sufficient material both from the time of initial diagnosis prior to treatment and at posttreatment recurrence. All 5 patients received combination chemotherapy including antimetabolites such as methotrexate between the initial diagnosis and recurrence. Patients with non-Hodgkin B cell lymphoma who did not receive antimetabolites were excluded from this study. None of the patients had known innate immunodeficiency or tumor predisposition syndromes. The UCSF Institutional Review Board approved this study.

### Immunofluorescence staining.

Immunostaining was performed on formalin-fixed, paraffin-embedded human lymphoma samples. In brief, the paraffin blocks were sliced into 5 μm–thick sections, deparaffinized with xylene substitute (Sigma), and rehydrated with decreasing concentrations of ethanol in water. For antigen retrieval tissue slides were incubated in 95°C sodium citrate buffer (pH 6.0) for 20 minutes in a steamer followed by 20 minutes of cooling at room temperature. Tissue was permeabilized in blocking buffer consisting of 0.4% Triton-X-100/10% normal goat serum/PBS for 30 minutes at room temperature. Overnight 1:100 diluted primary antibodies in PBS were applied at 4°C in a humidified chamber: anti-SLC15A3 antibody (Proteintech 20866). The next day, sections were washed 4 times with PBS with Tween (PBST) and incubated in secondary antibody (anti-rabbit 448 Invitrogen A21206, anti-mouse 568 Biotuim 20101) for 60 minutes at room temperature in a humidified chamber. Then sections were washed 4 times with PBST. The sections were mounted using ProLong Gold antifade reagent with DAPI (Invitrogen P36931). Stained sections were imaged using a digital microscope (Keyence BZ-X) and blindly quantified using Fiji ImageJ.

### Immunofluorescence and PLA fluorescence on cells.

Briefly, Ramos cells (ATCC, CRL-1596) or *Eμ-Myc/^+^* lymphoma cells were washed with PBS and seeded on Shi-fix coverslips (SB-SHIFIX50, Everest) in PBS for 20 minutes, followed by 1 wash with PBS to remove unattached cells. Cells were fixed with cold methanol for 5 minutes at –20°C, followed by 3 washes with PBS. For immunofluorescence, coverslips were blocked with 10% goat serum and incubated with primary antibodies (1:100) anti-SLC15A3 (Proteintech, 20866) and anti-mTOR (Cell Signaling Technology, L27D4) overnight at 4°C. Coverslips were washed twice using PBST at room temperature for 10 minutes. Coverslips were then incubated with secondary antibodies with fluorescent probes (Anti-mouse-567 [Biotium, 20101-1] and Anti-rabbit-488 [Invitrogen, A21206]) at room temperature for 1 hour from light. Coverslips were washed 3 times using PBST at room temperature for 10 minutes. Coverslips were then mounted to precleaned microscope slides (Thermo Fisher Scientific 12-550-15) using ProLong Gold antifade reagent with DAPI. Cells were visualized by confocal fluorescence microscopy using ZEISS Spinning Disk Confocal microscope.

PLA was performed as stated in the Duolink PLA Fluorescence Protocol (Sigma). Briefly, after fixation, blocking was done using the blocking buffer from Duolink flowPLA Detection Kit (Sigma) in a heated humidity chamber for 60 minutes at 37°C. Primary antibodies were diluted in Duolink Antibody Diluent — anti-SLC15A3 (1:100; Proteintech), anti-mTOR (1:100; Cell Signaling, L27D4) — and incubated overnight at 4°C. As a control, all antibodies were used alone (“none” conditions in figures) to quantify the background signal. All samples were probed with anti-mouse MINUS and anti-rabbit PLUS Duolink secondaries. Duolink in situ red reagents were used according to the manufacturer’s protocol (MilliporeSigma) and mounted with ProLong Gold antifade reagent with DAPI. To detect the colocalization with lysosome, after final PLA washes, cells were further blocked using Duolink PLA blocking solution. Cells were then stained for lysosomes using CoraLite 488 anti-LAMP2 (CL488-65053) in Duolink PLA 1× antibody dilution buffer overnight at 4°C while protected from light. Coverslips were washed twice using Duolink PLA Buffer B at room temperature for 10 minutes. Coverslips were washed once using 0.01× Duolink PLA Buffer B for 1 minute. Coverslips were then mounted to precleaned microscope slides (Thermo Fisher Scientific 12-550-15) using ProLong Gold antifade reagent with DAPI (P36931). cells were visualized by confocal fluorescence microscopy using Zeiss Spinning Disk Confocal microscope. The number of cellular PLA punctae were quantified and assessed for proximity to lysosomes. These data were analyzed using GraphPad Prism statistical analysis software.

### Quantitative PCR.

RNAs from lymphoma cells from mice or cell lines were isolated using RNeasy Mini Kit (QIAGEN). A total of 1 μg RNA was used as a template to synthesize single-stranded cDNA with the High-Capacity cDNA Reverse Transcription Kit (Applied Biosystems). cDNA samples were diluted 1:10 and 1 μL of template was used in a PowerUP SYBR Green Master Mix reaction run on an Applied Biosystems QuantStudio 6 Flex Real-Time PCR System (both Thermo Fisher Scientific). Oligonucleotide primers were based on either published literature or the National Center for Biotechnology Information site Primer-BLAST. Oligonucleotides for qPCR were as follows: Mouse SLC15A3-F: TGACCAGGTGATGGATCTCG, Mouse SLC15A3-R: ATGACACTTGGCTGCCTGTT, Mouse PRPS2-F: ATGAAGTGGACCGGATGGTT, Mouse PRPS2-R: GGTGGCACCAGCTGAGAGTA, Mouse SLC15A4-F: CGCCATCCTGCTCAGCC, Mouse SLC15A4-R: CGATCTTTAACCTGATCGGCG, Mouse 18S-F: GCAATTATTCCCCATGAACG, Mouse 18S-R: GGCCTCACTAAACCATCCAA, Human SLC15A3-F: TGGCGTTTATTCAGCAGAACA, Human SLC15A3-R: TCTCTGGCCGAGTGTCGTT, Human SLC15A4-F: GGACAAACTGGTCGATCCCAT, Human SLC15A4-R: AAAGGCCGAGCACATGACAA, Human 18S-F: AGTCCCTGCCCTTTGTACACA, Human 18S-R: GATCCGAGGGCCTCACTAAAC, Human PRPS1-F: ATCTTCTCCGGTCCTGCTATT, Human PRPS1-R: TGGTGACTACTACTGCCTCAAA, Human PRPS2-F: GGAGATTGGTGAAAGCGTGAG, Human PRPS2-R: AGGTTGTCGTTAATTTCCCCG, Human SLC38A9-F: CAGTGGTCGAGTCTCCTTTTC, Human SLC38A9-R: TCCCGGCACTTGGACAAATC, Human Sestrin2-F: AAGGACTACCTGCGGTTCG, Human Sestrin2-R: CGCCCAGAGGACATCAGTG, Human CAMP-F: AGGTCCTCAGCTACAAGGAAG, and Human CAMP-R: TCTTGAAGTCACAATCCTCTGGT.

### Cell culture.

Ramos cells were cultured in RPMI-1640 medium supplemented with 10% FBS and 100 U/mL penicillin and 100 μg/mL streptomycin. Mouse Eμ-*Myc*/^+^ lymphoma cells (19) were a gift from Luke Gilbert (UCSF) and were cultured in 45% DMEM/45% IMDM/10% FBS, supplemented with 2 mM l-glutamine, 50 μM β-mercaptoethanol, and 100 U/mL penicillin and 100 μg/mL streptomycin. To generate resistant cells, Ramos cells or Eμ-*Myc*/^+^ lymphoma cells were treated in the presence of 10 nM 6-MP Monohydrate (Selleckchem), with the concentration gradually increased to 2 μM over 2 months. To monitor cell survival, cell numbers were quantified using CellTiter-Glo assay (Promega G7570) following manufacturer’s instructions with luminescence measurements made using a Glomax 96-well plate luminometer (Promega).

Cells were incubated in HBSS with vehicle or different dipeptides for 2 hours. Then CellTiter-Glo assay was performed following manufacturer’s instructions with luminescence measurements made using a Glomax 96-well plate luminometer.

### shRNA knockdown and cDNA overexpression.

The pLKO.1 (Addgene, catalog 10878) backbone was used for shRNA knockdown. For pLKO.1 shRNA hairpin cloning, sense and antisense insert oligonucleotides were annealed and ligated into the cut backbone at room temperature for at least 1 hour using T4 DNA ligase. Lentivirus expressing shRNA or cDNA (Origene SLC15A3 NM_016582) was prepared using HEK293T cells (ATCC, CRL-3216). Briefly, HEK293T cells were seeded in a 10 cm dish and transfected with 1.5 μg pMD2.G (Addgene, no. 12259), 3.5 μg PsPAX2 (Addgene, no. 12260), and 5 μg human pLKO.1 with different shRNAs using Lipofectamine 2000 (Thermo Fisher Scientific, no. 11668027). The viral supernatant was collected at 48 hours posttransfection. Viral particles were concentrated by Lenti-X Concentrator (TaKaRa, 631231), resuspended with DPBS, and immediately stored at –80°C in single-use aliquots. Lentiviral particles were added dropwise to 300,000 cells per well in a 12-well plate containing 1.5 mL of medium supplemented with 4 μg/mL polybrene. The plate was centrifuged at 800*g* for 1 hour at room temperature (spinfection). Following centrifugation, cells were incubated overnight at 37°C. The next day, the medium was replaced with fresh culture medium. Puromycin selection (Thermo Fisher Scientific, no. A1113802) was initiated 48–72 hours postinfection to select for transduced cells. After 72 hours of being selected with puromycin, the cells were collected and split for protein and RNA expression analyses. The hairpin sequences are listed here: Mouse_SLC15A3_1_f:CCGGGCCTTATTGGATGGTATATTTCTCGAGAAATATACCATCCAATAAGGCTTTTTG, Mouse_SLC15A3_1_r:AATTCAAAAAGCCTTATTGGATGGTATATTTCTCGAGAAATATACCATCCAATAAGGC, Mouse_SLC15A3_2_f:CCGGCGCTTCTTCAACTGGTTCTATCTCGAGATAGAACCAGTTGAAGAAGCGTTTTTG, Mouse_SLC15A3_2_r:AATTCAAAAACGCTTCTTCAACTGGTTCTATCTCGAGATAGAACCAGTTGAAGAAGCG, Human_SLC15A3_1_f:CCGGGACCGCTTGATCGACCCTTTACTCGAGTAAAGGGTCGATCAAGCGGTCTTTTTG, Human_SLC15A3_1_r:AATTCAAAAAGACCGCTTGATCGACCCTTTACTCGAGTAAAGGGTCGATCAAGCGGTC, hSLC38A9_shRNA2_f:CCGGGCCTTGACAACAGTTCTATATCTCGAGATATAGAACTGTTGTCAAGGCTTTTTG, hSLC38A9_shRNA2_r:AATTCAAAAAGCCTTGACAACAGTTCTATATCTCGAGATATAGAACTGTTGTCAAGGC, hSESN2_shRNA2_f:CCGGGAAGACCCTACTTTCGGATATCTCGAGATATCCGAAAGTAGGGTCTTCTTTTTG, hSESN2_shRNA2_r:AATTCAAAAAGAAGACCCTACTTTCGGATATCTCGAGATATCCGAAAGTAGGGTCTTC.

### Dipeptide tracing experiment.

Cells were treated with labeled dipeptides [^13^C-glycine-(N15) leucine, ^13^C-leucine-(N15) glutamine (CIL)] in full medium for 24 hours, quickly washed with ice-cold normal saline solution, and added to 1 mL of 80% (vol/vol) methanol (cooled to −80°C). Cell lysate/methanol mixture was then incubated at −80°C for 20 minutes and transferred to a 1 mL Eppendorf tube and frozen in liquid nitrogen.

Data acquisition was performed by reverse-phase chromatography on a 1290 UHPLC liquid chromatography system interfaced to a high-resolution mass spectrometry 6550 iFunnel Q-TOF mass spectrometer (Agilent Technologies). The MS was operated in both electrospray ion–positive and –negative (ESI^+^ and ESI^–^) modes. Analytes were separated on a Waters Acquity UPLC HSS T3 column (1.8 μm, 2.1 × 150 mm). The column was kept at room temperature. Mobile phase A composition was 0.1% formic acid in water, and mobile phase B composition was 0.1% formic acid in 100% acetonitrile. The LC gradient was 0 minutes: 1% B; 5 minutes: 5% B; 15 minutes: 99% B; 23 minutes: 99% B; 24 minutes: 1% B; 25 minutes: 1% B. The flow rate was 250 μL/min. The sample injection volume was 5 μL. ESI source conditions were set as follows: dry gas temperature 225°C and flow 18 L/min, fragmentor voltage 175 V, sheath gas temperature 350°C and flow 12 L/min, nozzle voltage 500 V, and capillary voltage +3,500 V in positive mode and −3,500 V in negative. The instrument was set to acquire over the full *m/z* range of 40–1,700 in both modes, with the MS acquisition rate of 1 spectrum s-1 in profile format.

Raw data files (.d) were processed using isotopic extraction in Profinder B.08.00 SP3 software (Agilent Technologies) with an in-house database containing retention time and accurate mass information on 600 standards from Mass Spectrometry Metabolite Library (IROA Technologies), which was created under the same analysis conditions. The in-house database matching parameters were mass tolerance 10 ppm, retention time tolerance 0.5 minutes, retention time correlation coefficient 0.5.

### Ribo-seq and RNA-seq.

Lymphoma cells were harvested from tumor-bearing *Eμ-Myc/^+^* mice and *Eμ-Myc/^+^ PRPS2^null^* mice as described before. A small portion of the samples were used to prepare Ribo-seq libraries, using the published protocol (45, 46) with some alterations described below. Lymphoma cells were lysed in 20 mM Tris pH 7.5, 150 mM NaCl, 5 mM MgCl_2_, 1 mM DTT, 100 μg/mL cyclohexamide, 1% Triton X-100, and 25 U/mL Turbo DNase I on ice for 30 minutes by vortexing 3 times. Lysates were spun down at 10,000*g* for 10 minutes, and the RNA concentration was measured by Qubit RNA HS Assay kit (Invitrogen). Respectively, 30 μg and 1 μg of RNA containing lysates were used to process Ribo-seq and RNA-seq libraries. Then 30 μg RNA containing lysate was digested with 1.5 μL RNase I (10 U/μL by the Epicentre definition) and incubated for 45 minutes at room temperature with gentle agitation. RNase digestion was inhibited by adding 10 μL SuperaseIN RNase Inhibitor and immediately putting the samples on ice. Ribosomes with footprints were isolated using MicroSpin S-400 column. RNAs were isolated by mixing with TRizol-LS and precipitated by isopropanol and glycogen. Next, purified RNA samples were run on a 15% TBE-urea gel, then stained by SyberGold, and the bands above 24 nt and below 34 nt were extracted from the gel. Dephosphorylation and linker ligation of the footprint fragments followed by reverse transcription, circularization of cDNA, and the library construction were prepared as described (45).

For the RNA-seq libraries, RNA was isolated by TRIzol from the lymphoma lysates. Only 10 ng of total RNA was used following the TaKaRa SMARTer Stranded Total RNA-Seq kit protocol, and both libraries were submitted to the UCSF Center for Advanced Technology (CAT) facility.

For Ribo-seq analysis, to process the reads, the Ribo-seq reads were first trimmed using cutadapt (v2.3) to remove the linker sequence AGATCGGAAGAGCAC. The fastx_barcode_splitter script from the Fastx toolkit (v0.0.13) was then used to split the samples based on their barcodes. Since the reads contain unique molecular identifiers (UMIs), they were collapsed to retain only unique reads. The UMIs were then removed from the beginning and end of each read (2 and 5 nucleotides, respectively) and appended to the name of each read. Bowtie2 (v2.3.5) was then used to remove reads that mapped to ribosomal RNAs and tRNAs, and the remainder of the reads were then aligned to mRNAs (we used the isoform with the longest coding sequence (CDS) for each gene as the representative). After alignment, umitools (v0.3.3) was used to deduplicate reads. For the associated total RNA-seq, reads were aligned to the longest CDS using Bowtie2. Ribo-seq and RNA-seq reads (BAM files) were then input into Ribolog (47). For the Ribo-seq data, reads between 23 and 32 nt in length were selected. The P-site offsites and codon counts were calculated for each sample using *psite_info* and *psite_to_codon_count* functions, respectively adapted from the riboWaltz package in R (48). Codon counts were corrected for stalling using the *CELP_bias* function from the *Ribolog* package in *R*. Translation efficiency (TE) ratios for each transcript were calculated as the ratio between (median of ratios-normalized) Ribo-seq/total RNA-seq transcript counts. TE changes between conditions were modeled using logistic regression, specifically utilizing the function *logit_seq* in the *Ribolog* package.

### Identification of TOP motifs in translationally upregulated mRNAs.

To identify regulatory motifs associated with translational regulation, 5′ UTR sequences were extracted from the reference transcriptome (35–37) for all mRNAs in the ribosome profiling dataset. 5′TOP motifs were identified using regular expression pattern matching, with the TOP motif defined as a cytosine at the +1 position followed by 4–15 consecutive pyrimidines (49, 50). mRNAs were then stratified into 2 groups: translationally upregulated (logTER > 1, *P* adjusted value < 0.05 in ribosome profiling data) and all other mRNAs. Fisher’s exact test was performed to assess enrichment of TOP motifs in the translationally upregulated mRNAs relative to all other mRNAs, with statistical significance defined as a *P* value < 0.05.

### Analysis of VDR:RXRA binding sites in the SLC15A3 promoter.

Candidate transcription factors associated with the *SLC15A3* promoter were identified using Enrichr (51) with the JASPAR PWM Human 2025 library, which identified RXRA:VDR as a top candidate (Supplemental Table 5 and Supplemental Figure 6C). The VDR:RXRA heterodimer binding position weight matrix (MA0074.1) was obtained from the JASPAR 2025 database (52). To evaluate potential binding sites, promoter sequences extending 2,000 bp upstream of the TSS were extracted for all annotated SLC15A3 transcript isoforms from Ensembl release 114 (53). Motif scanning along the SLC15A3 promoter sequences was performed using FIMO (v5.5.7) (54) from the MEME Suite with a *P* value threshold of 1 × 10^–4^.

### Dipeptide uptake assay.

Cells were treated with different concentrations of d-Ala-Lys-AMCA (MedChemExpress) for 2 hours. Then the cells were washed once with PBS, and the intensity of AMCA was measured using flow cytometry or plate reader (excitation/emission = 390/480 nm). Cells were further lysed using RIPA buffer, and cell lysates were measured using plate reader (excitation/emission = 390/480 nm), and protein concentration was measured using BCA assay.

### Acridine orange staining.

Cells were resuspended in prewarmed complete medium and stained with acridine orange (1 μg/mL) for 15 minutes at 37°C protected from light. Cells were washed twice with PBS and analyzed immediately by flow cytometry. After gating on live singlets, lysosomal acidification was quantified as the red/green fluorescence ratio (55).

### Statistics.

All values in the text and figures are presented as the means ± SEM. The differences were analyzed using unpaired 2-sided Student’s *t* test, 1-way ANOVA, 2-way ANOVA, log-rank (Mantel-Cox) test, and simple linear regression indicated in figure legends and source files. All statistical analyses were performed using GraphPad Prism software. For all statistical analyses, *P* values are indicated in each figure panel and source files (not significant, *P* > 0.05). All the experimental findings were reliably reproduced as validated by at least 3 biological independent replicates.

### Study approval.

All animal procedures were approved by the University of California, San Francisco, Institutional Animal Care and Use Committee. Mice were maintained under pathogen-free conditions, and all experiments were performed in compliance with guidelines approved by the Institutional Animal Care and Use Committee of UCSF. Patient sample research and procedures were approved by the UCSF Institutional Review Board (IRB). IRB (18-25787) waived patient consent given the minimal study risk.

### Data availability.

All data necessary for the conclusions of the study are provided with the article and in Supporting Data Values. Sequencing data generated for the Ribo-seq and RNA-seq analysis of lymphoma tumor from Eμ-*Myc/^+^* mice and Eμ-*Myc/^+^ Prprs2*^null^ mice are available at the Gene Expression Omnibus under SuperSeries accession number GSE328313.

## Author contributions

HY and DR designed the experimental outline and wrote the manuscript. DR supervised the project. HY, VAZ, and KB performed experiments. EG, LX, SM, HV, KW, HGN, RJD, AN, SC, HG, SH, and IL assisted with experiments, analyzed, and provided research expertise.

## Conflict of interest

DR is a Scientific Advisory Board member at SJP Biotec and MelliCell. RJD is a founder and advisor at Atavistik Bioscience and an advisor at Vida Ventures, Faeth Therapeutics, and Illumina.

## Funding support

This work is the result of NIH funding, in whole or in part, and is subject to the NIH Public Access Policy. Through acceptance of this federal funding, the NIH has been given a right to make the work publicly available in PubMed Central.

- American Heart Association (P0564550) (to HY).
- NIH (K99AG087723) (to HY).
- HHMI Investigator Program and the NIH (R35CA220449) (to RJD).
- Children’s Research Institute Metabolomics Core, supported in part by CPRIT (RP240494) (to DR).
- NIH (R35CA242986) (to DR).
- American Cancer Society (American Cancer Society Research Professor Award) (to DR).
- NIH (T32CA151022) (to EG).
- Weill Award for Clinician-Scientists in the Neurosciences, Weill Institute for Neurosciences (to EG).
- Experimental Neuropathology Endowment Award, UCSF (to EG).
- Department of Defense W81XWH-2210121 (to HG).

## Supplementary Material

Supplemental data

Unedited blot and gel images

Supplemental table 1

Supplemental table 2

Supplemental table 3

Supplemental table 4

Supplemental table 5

Supplemental table 6

Supplemental table 7

Supporting data values

## Version 1 07/15/2026

Electronic publication
