# Supplementary material for: SLC15A3-mediated dipeptide metabolism confers antimetabolite resistance in lymphoma via mTORC1 activation
Source: J Clin Invest. 2026 Jul 15;136(14):e199709. doi: 10.1172/JCI199709 (PMC13367966; doi:10.1172/JCI199709)
Supplement: Supplemental data [file jci-136-199709-s040.pdf]

Supplementary Figures for

SLC15A3-Mediated Dipeptide Metabolism Confers Antimetabolite Resistance in Lymphoma via mTORC1 Activation

Haojun Yang[1,2,3‡], Vincenzo Andrea Zingaro[1,2], Kevin Boardman[1,2], Ashish Noronha[1,2], Ekin Guney[1,2,4,5], Lingru Xue[1,2], Saishma Hoigebazar[1,2], Isabelle Liu[1,2,6], Sohit Miglani[1,2], Siyu Chen[6,7], Hieu Vu[8], Kwun Wah Wen[1,4], Hao Nguyen[1,2], Hani Goodarzi [6,7], Ralph J. DeBerardinis[9,10], Davide Ruggero[1,2,11‡]

Affiliations:

[1]Helen Diller Family Comprehensive Cancer Center, UCSF, San Francisco, CA, US

[2]School of Medicine and Department of Urology, UCSF, San Francisco, CA, USA

[3]School of Medicine and Department of Pharmacological Sciences, Stony Brook University, Stony Brook, NY, USA

[4]Department of Pathology, UCSF, San Francisco, CA, USA

[5]Department of Neurology, UCSF, San Francisco, CA, USA

[6]Department of Biochemistry & Biophysics, UCSF, San Francisco, CA, USA

[7]Arc Institute, Palo Alto, California, USA

[8]Department of Pathology and Center of Excellence for Leukemia Studies, St. Jude Children's Research Hospital, Memphis, TN, USA

[9]Eugene McDermott Center for Human Growth and Development, Children's Research Institute, Department of Pediatrics, University of Texas Southwestern Medical Center, Dallas, TX, USA.

[10]Howard Hughes Medical Institute, UT Southwestern Medical Center, Dallas, TX, USA.

[11]Department of Cellular and Molecular Pharmacology, UCSF, CA, USA

ǂ Correspondence

Address correspondence to: Davide Ruggero, 1450 3rd St, San Francisco, CA 94158; Email: Davide.Ruggero@ucsf.edu.

Or to: Haojun Yang, 101 Nicolls Rd, Stony Brook, NY 11794; Email: Haojun.Yang@stonybrook.edu

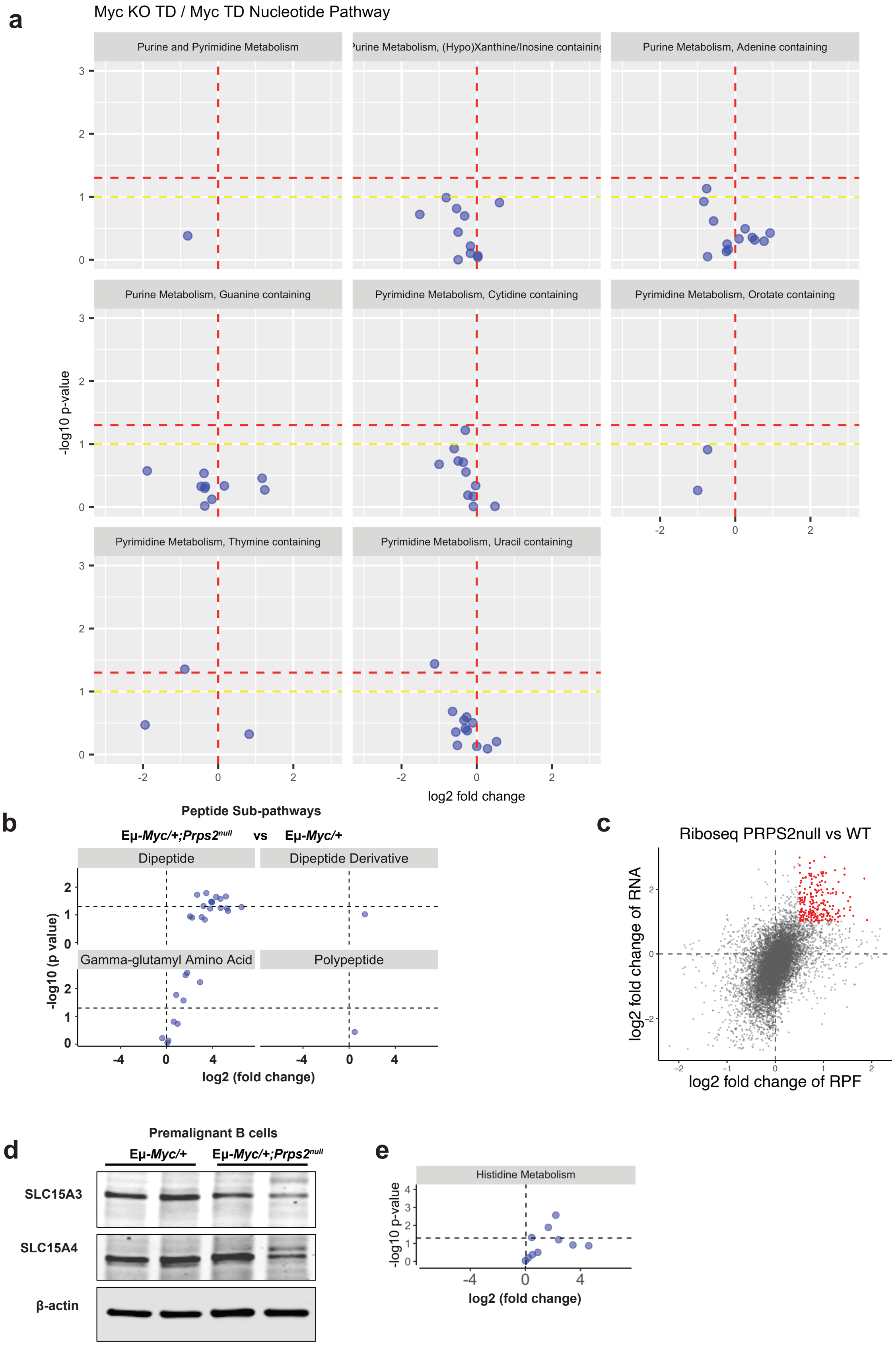
a
Myc KO TD / Myc TD Nucleotide Pathway
Purine and Pyrimidine Metabolism
Purine Metabolism, (Hypo)Xanthine/Inosine containing
Purine Metabolism, Adenine containing
Purine Metabolism, Guanine containing
Pyrimidine Metabolism, Cytidine containing
Pyrimidine Metabolism, Orotate containing
Pyrimidine Metabolism, Thymine containing
Pyrimidine Metabolism, Uracil containing
-log10 p-value
log2 fold change
b
Peptide Sub-pathways
Eμ-Myc/+;Prps2null vs Eμ-Myc/+
Dipeptide
Dipeptide Derivative
Gamma-glutamyl Amino Acid
Polypeptide
-log10 (p value)
log2 (fold change)
c
Riboseq PRPS2null vs WT
log2 fold change of RNA
log2 fold change of RPF
d
Premalignant B cells
Eμ-Myc/+
Eμ-Myc/+;Prps2null
SLC15A3
SLC15A4
β-actin
e
Histidine Metabolism
-log10 p-value
log2 (fold change)

Supplemental Figure 1. Nucleotide-deficiency resistant large B cell lymphoma specifically upregulates dipeptides and their transporter SLC15A3.

a. Volcano plot of metabolites comparing lymphoma from Eμ-*Myc/+; Prps2*^{null} (Myc KO TD) mice to Eμ-*Myc/+* (Myc TD) mice in each sub-pathway within nucleotide pathway, and **b.** in each sub-pathway within peptide pathway. **c.** Two-dimensional plot of log2 fold change in gene expression at the translational (RPF: ribosome-protected fragments, x-axis) and RNA (y-axis) levels in lymphomas from Eμ-*Myc/+; Prps2*^{null} and Eμ-*Myc/+* mice. Red points indicate genes upregulated at both transcriptional and translational levels. **d.** Immunoblot analysis of indicated proteins in B cells from Eμ-*Myc/+* and Eμ-*Myc/+; Prps2*^{null} mice. β-actin serves as the loading control. **e.** Volcano plot of metabolites comparing lymphoma from Eμ-*Myc/+; Prps2*^{null} mice to Eμ-*Myc/+* mice in histidine metabolism sub-pathway.

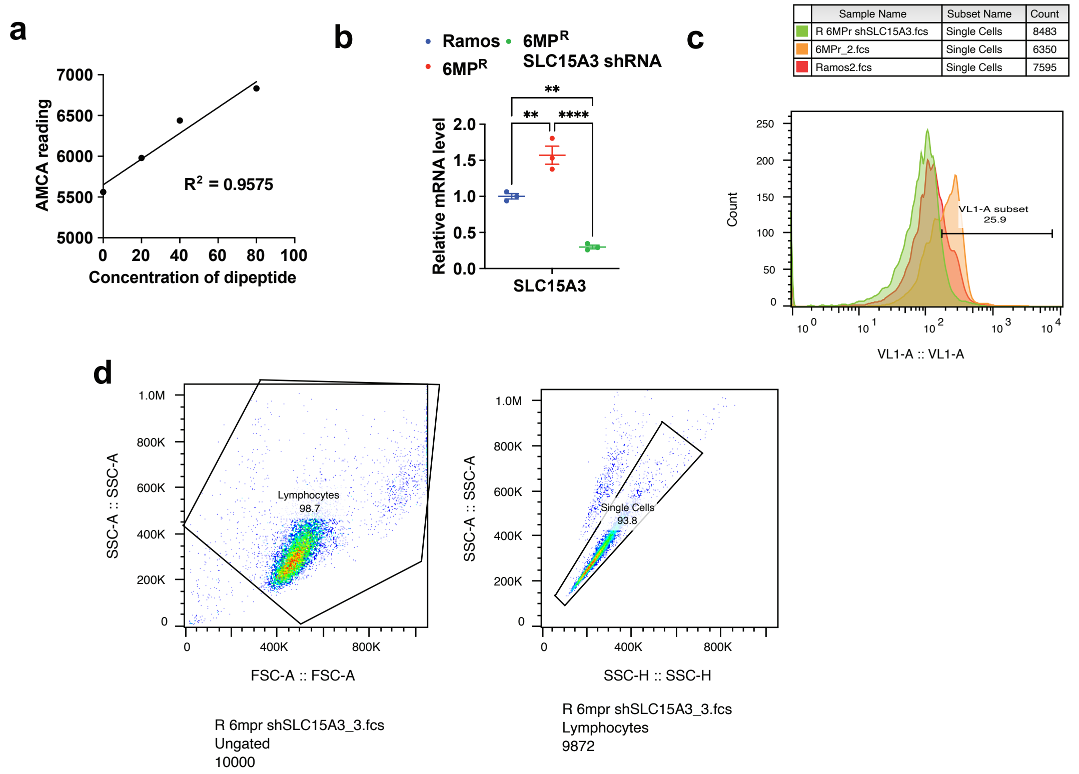

Supplemental Figure 2. 6MP-resistant human lymphomas upregulate dipeptide uptake and SLC15A3.

a. Standard curve of AMCA reading in the lysates of cells incubated with different concentrations of AMCA labeled dipeptide. **b.** Relative mRNA levels of *SLC15A3* in parental and 6MP-resistant Ramos cells without or with shRNA-mediated knockdown of *SLC15A3*. **c.** Representative flow cytometric analysis of dipeptide-AMCA uptake in parental and 6MP-resistant Ramos without or with shRNA-mediated knockdown of *SLC15A3.* **d.** Gating strategies for flow cytometric analysis of dipeptide-AMCA in Ramos cells.

Individual data and mean ± SEM were shown in a and b, a was analyzed using simple linear regression, b was analyzed using 1-way ANOVA, **P < 0.01, ****P < 0.0001.

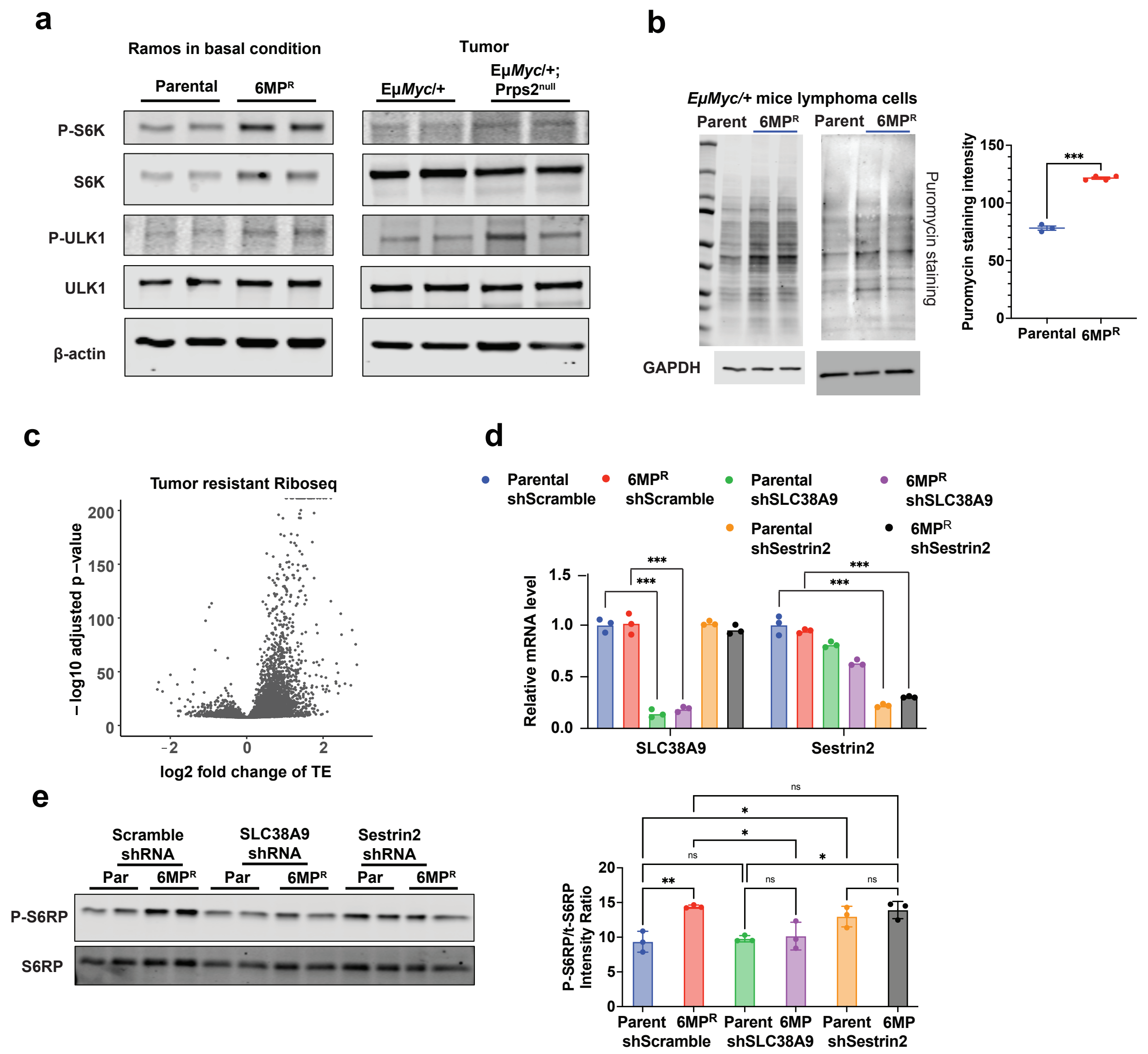

Supplemental Figure 3. Elevated dipeptides are associated with enhanced mTORC1 activation in resistant lymphomas.

a. Immunoblot analysis of indicated proteins in parental or 6MP-resistant Ramos cells under full medium condition, and in Eμ-*Myc*/+; *Prps2*^{null} and Eμ-*Myc*/+ lymphomas. **b.** Immunoblot analysis and quantification of puromycin incorporation for 30 min in parental and 6MP-resistant Eμ-*Myc*/+ cells under basal condition, GAPDH serves as the loading control. **c.** Volcano plot of log2 fold change of translation efficiency (TE) and -log10 of p-value of translation efficiency of genes when comparing Eμ-*Myc/+; Prps2*^{null} and Eμ-*Myc/+* lymphoma. **d.** Relative mRNA levels of *SLC38A9* and *SESTRIN2* in parental and 6MP-resistant Ramos cells without or with shRNA-mediated knockdown of *SLC38A9* or *SESTRIN2*. **e.** Immunoblot analysis of indicated proteins in parental and 6MP-resistant Ramos cells without or with shRNA-mediated knockdown of *SLC38A9* or *SESTRIN2,* and the quantification of P-S6RP/t-S6RP ratio.

Individual data and mean ± SEM were shown in b, d, and e, and b was analyzed using t-test, d was analyzed using 2-way ANOVA, e was analyzed using 1-way ANOVA, ns $P > 0.05$, *$P < 0.05$, **$P < 0.01$; ***$P < 0.001$; ****$P < 0.0001$.

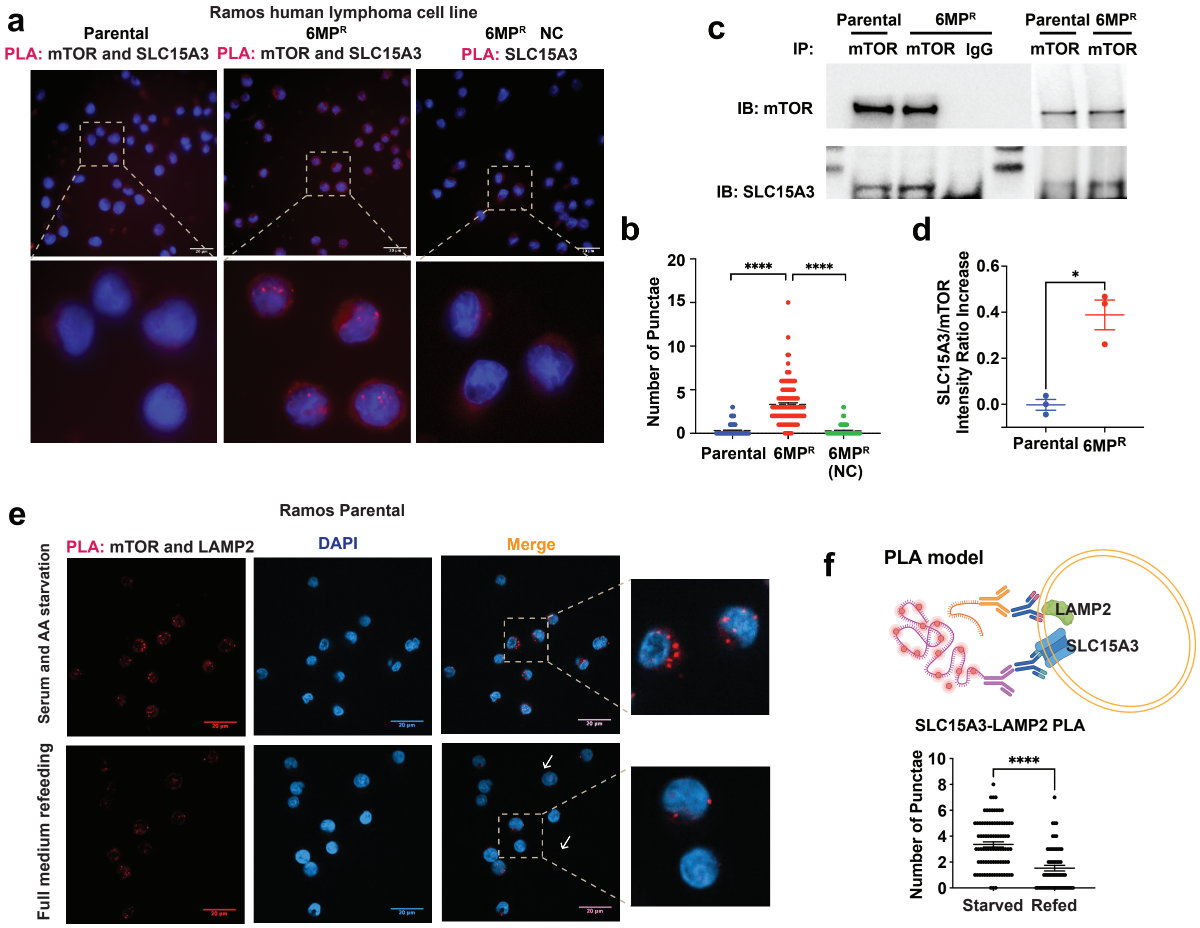

Supplemental Figure 4. SLC15A3 co-localizes with mTOR in 6MP-resistant lymphoma.
a. Representative images and **b.** quantification of proximity ligation assay (PLA) staining (red) using mTOR and SLC15A3 antibodies in parental and 6MP-resistant Ramos cells. PLA staining using only the SLC15A3 antibody in 6MP-resistant cells serves as a negative control. **c.** Immunoblot of co-IP using mTOR antibody or IgG in parental and 6MP-resistant Ramos cells. **d.** Relative increase of pull-down intensity ratio of SLC15A3 compared to mTOR in parental and 6MP-resistant Ramos cells**. e.** Representative images and **f.** quantification of proximity ligation assay (PLA) staining (red) using LAMP2 and SLC15A3 antibodies in Ramos cells after 45 min amino acid starvation and 15 min refeeding with full medium.
Individual data and mean ± SEM were shown in d and f were analyzed using t-test, b was analyzed using 1-way ANOVA, *P < 0.05, ****P < 0.0001.

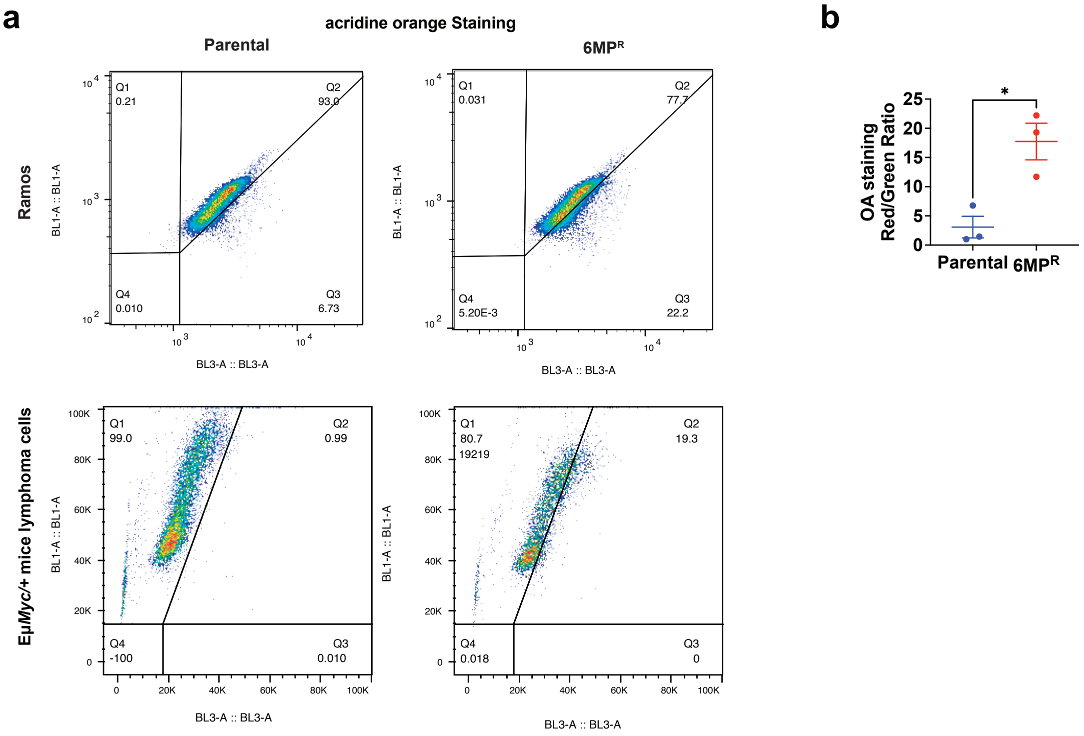

Supplemental Figure 5. Enhanced lysosome acidification in 6MP-resistant cells.
a. Representative flow cytometric analysis of red and green fluorescence in Acridine Orange–stained cells, displayed using a red/green ratio–based thresholding strategy. **b.** Quantification of the red/green fluorescence ratio, corresponding to Q3 in the upper panel of **a** and Q2 in the lower panel of **a**.
Individual data and mean ± SEM were shown in b, and analyzed using t-test, $*P < 0.05$.

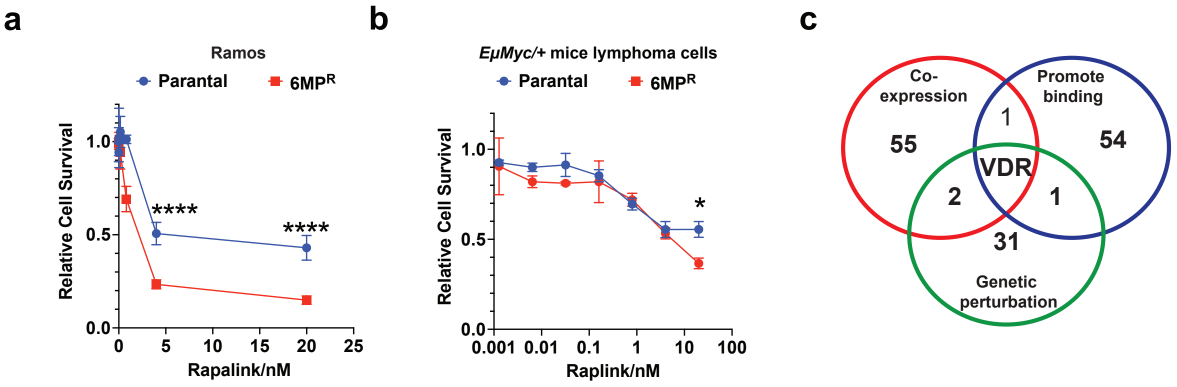

d

VDR response elements

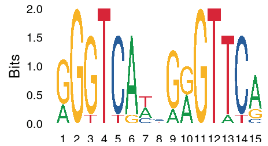

Motif ID	Alt ID	Sequence Name	Strand	Start	End	p-value	q-value	Matched Sequence
MA0074.1	RXRA::VDR	SLC15A3_ENST00000541505	-	1363	1377	8.62e-06	0.0683	GGGTCACAGGGCTCC
MA0074.1	RXRA::VDR	SLC15A3_ENST00000681015	-	1464	1478	8.62e-06	0.0683	GGGTCACAGGGCTCC
MA0074.1	RXRA::VDR	SLC15A3_ENST00000681275	-	1797	1811	8.62e-06	0.0683	GGGTCACAGGGCTCC
MA0074.1	RXRA::VDR	SLC15A3_ENST00000679573	-	1893	1907	8.62e-06	0.0683	GGGTCACAGGGCTCC
MA0074.1	RXRA::VDR	SLC15A3_ENST00000227880	-	1893	1907	8.62e-06	0.0683	GGGTCACAGGGCTCC
MA0074.1	RXRA::VDR	SLC15A3_ENST00000681951	-	1893	1907	8.62e-06	0.0683	GGGTCACAGGGCTCC
MA0074.1	RXRA::VDR	SLC15A3_ENST00000681882	-	1893	1907	8.62e-06	0.0683	GGGTCACAGGGCTCC
MA0074.1	RXRA::VDR	SLC15A3_ENST00000536491	-	918	932	1.11e-05	0.0683	AGGTCAATAGGGTCA
MA0074.1	RXRA::VDR	SLC15A3_ENST00000536784	-	943	957	1.11e-05	0.0683	AGGTCAATAGGGTCA

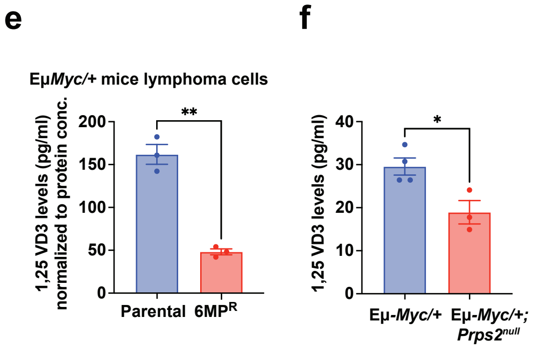

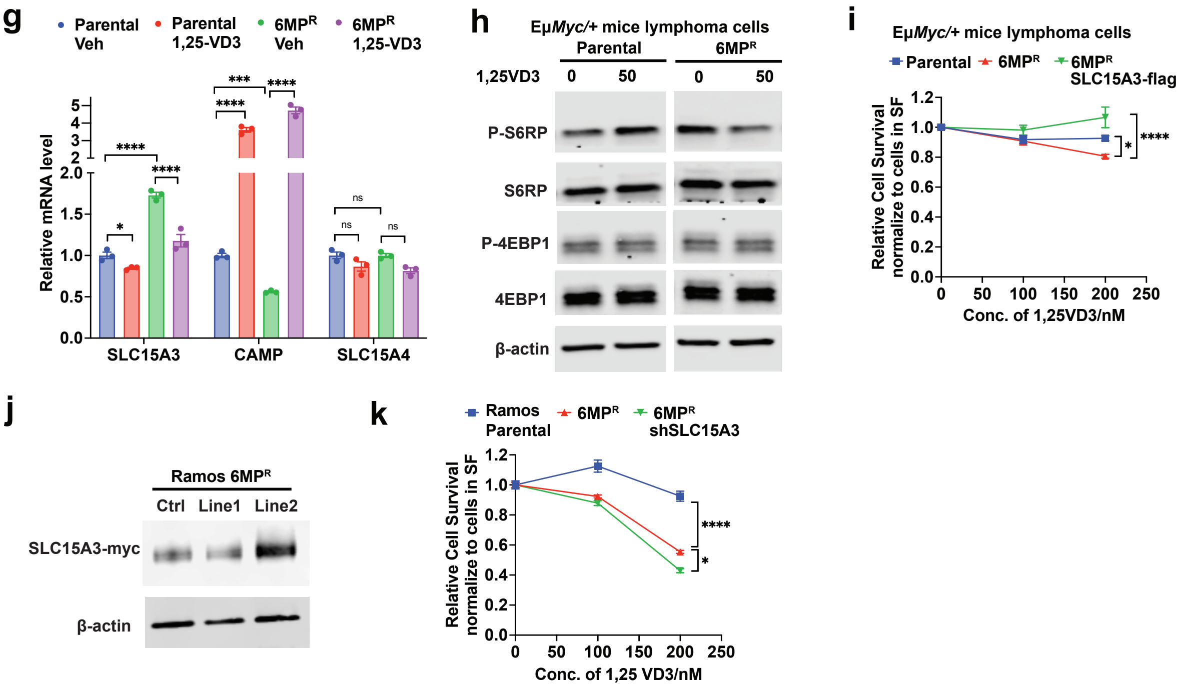

Supplemental Figure 6. Resistant lymphomas are more sensitive to rapamycin and active VD3 in vitro and in vivo.

a. Relative cell survival of parental and 6MP-resistant Ramos cells treated with different concentrations of Rapalink for 2 days. **b.** Relative cell survival of parental and 6MP-resistant Eμ-*Myc*/+ lymphoma cells treated with different concentrations of Rapalink for 2 days. **c.** Venn diagram showing the overlap of genes identified by co-expression analysis, promoter binding, and genetic perturbation. **d.** VDR response element motif and significant matched sequences identified at the upstream of SLC15A3 promoter region. **e.** Cellular 1,25-VD3 levels normalized by protein levels in parental and 6MP-resistant Eμ-*Myc*/+ lymphoma cells. **f.** Cellular 1,25-VD3 levels normalized by protein levels in Eμ-*Myc*/+ or Eμ-*Myc*/+;*Prps2*^{null} lymphomas. **g.** Relative mRNA levels of SLC15A3, SLC15A4 and VDR downstream target CAMP in parental (blue) and 6MP-resistant Ramos cells (red) treated with 100 nM 1,25-VD3 for 24h. **h.** Immunoblot analysis of indicated proteins in parental or 6MP-resistant Eμ-*Myc*/+ lymphoma cells treated with 0 or 50 nM 1,25-VD3 for 24 h. **i.** Relative cell survival of parental and 6MP-resistant Eμ-*Myc*/+ lymphoma cells without and with overexpression of SLC15A3 cDNA (line2) treated with different concentrations of 1,25-VD3 for 2 days. **j.** Immunoblot analysis of indicated proteins in 6MP-resistant Ramos cells infected without or with SLC15A3 cDNA lentivirus (line1, line2). **k.** Relative cell survival of parental and 6MP-resistant Ramos cells, with or without shSLC15A3, following treatment with different concentrations of 1,25-VD3 for 2 days.

Individual data and mean ± SEM were shown in a, b, e, f, g, i and k, and e, e and f were analyzed using t-test, a, b, g, i and k were analyzed using 2-way ANOVA, *P < 0.05, **P < 0.01; ****P < 0.0001.
