# Supplementary material for: SLC15A3-mediated dipeptide metabolism confers antimetabolite resistance in lymphoma via mTORC1 activation
Source: J Clin Invest. 2026 Jul 15;136(14):e199709. doi: 10.1172/JCI199709 (PMC13367966; doi:10.1172/JCI199709)

Figure 1f

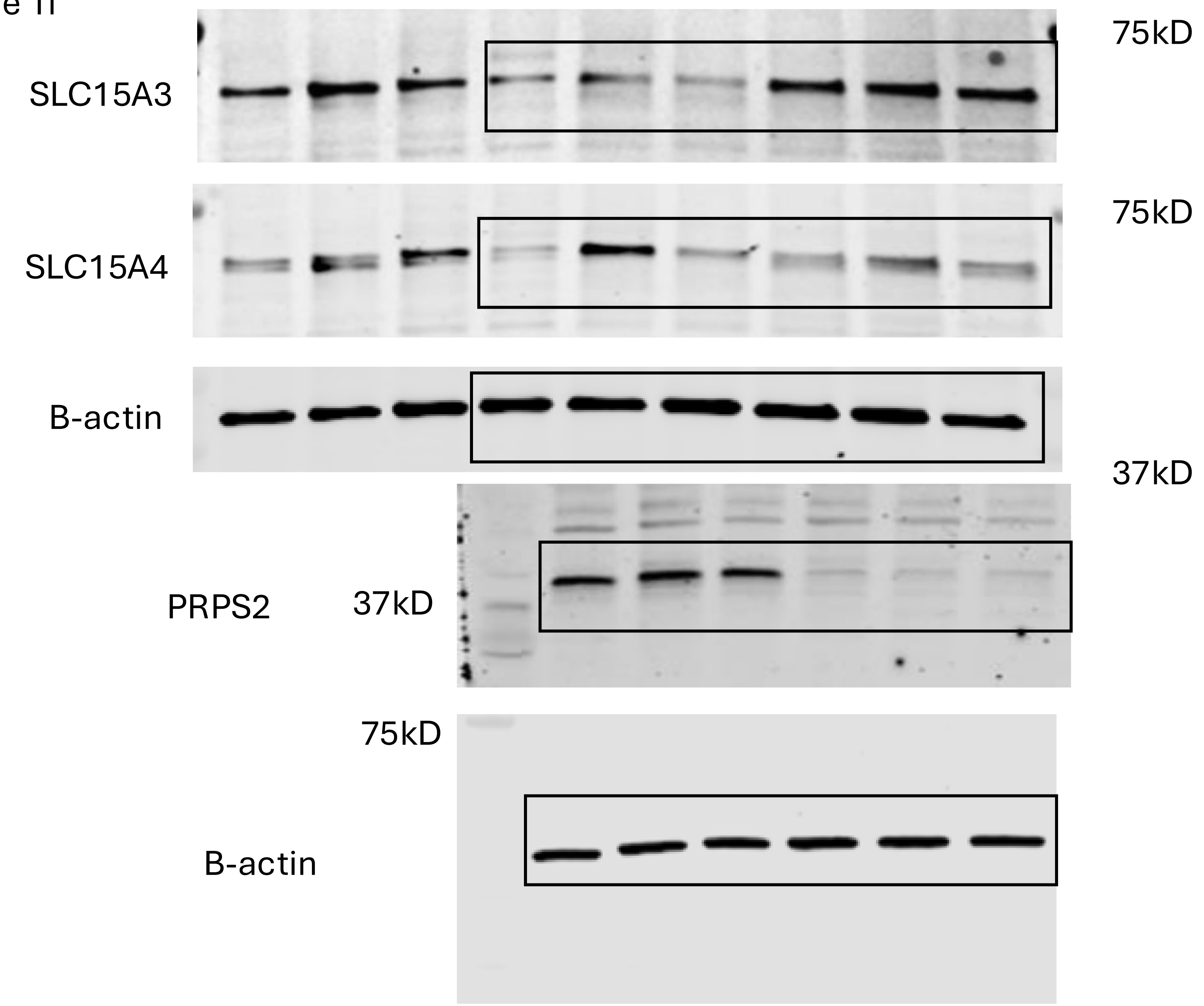
SLC15A3
75kD
SLC15A4
75kD
B-actin
37kD
PRPS2
37kD
75kD
B-actin

Figure 2d

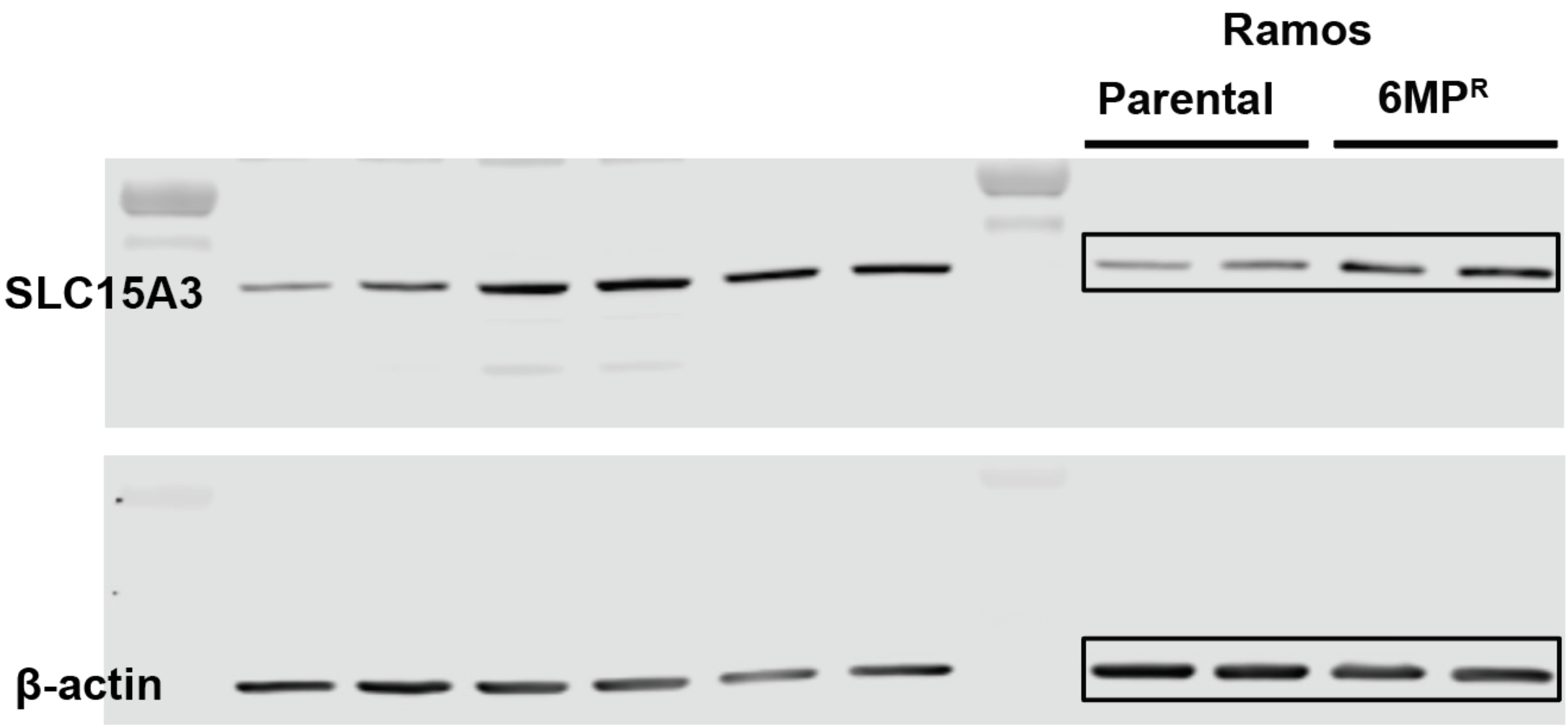

Figure 3e

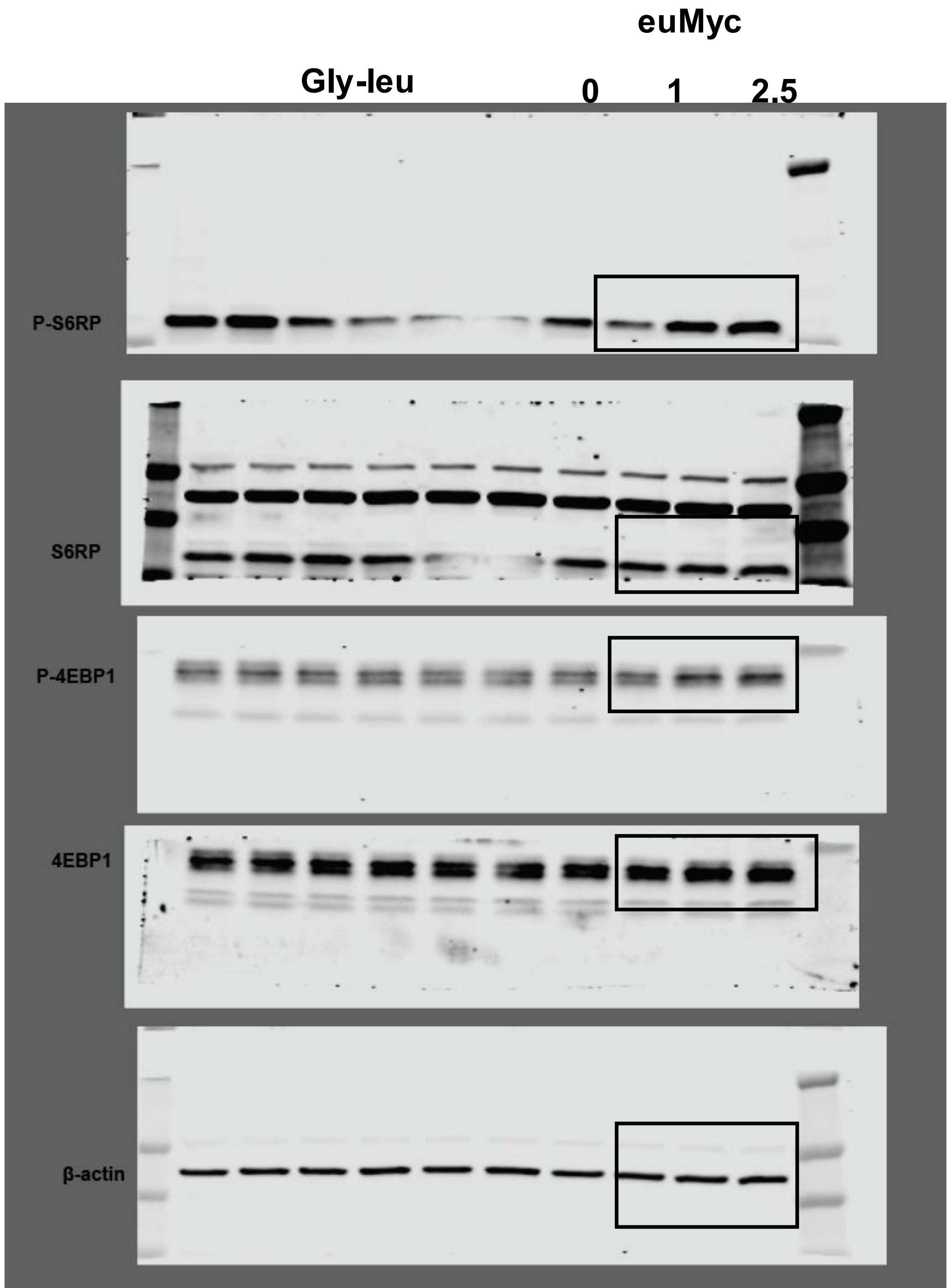

Figure 3f

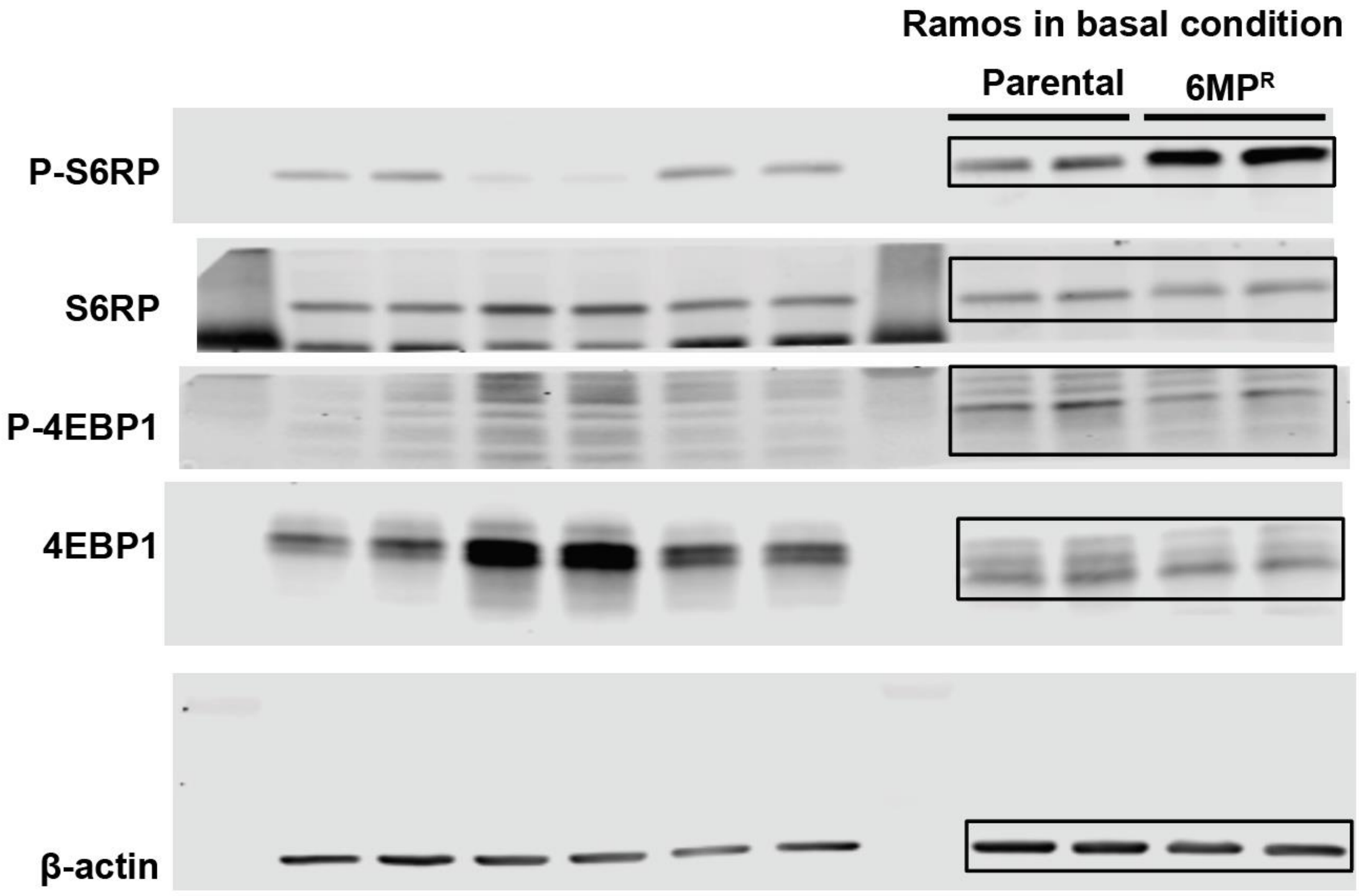

Ramos in basal condition
Parental
6MP^R
P-S6RP
S6RP
P-4EBP1
4EBP1
β-actin

Figure 3h

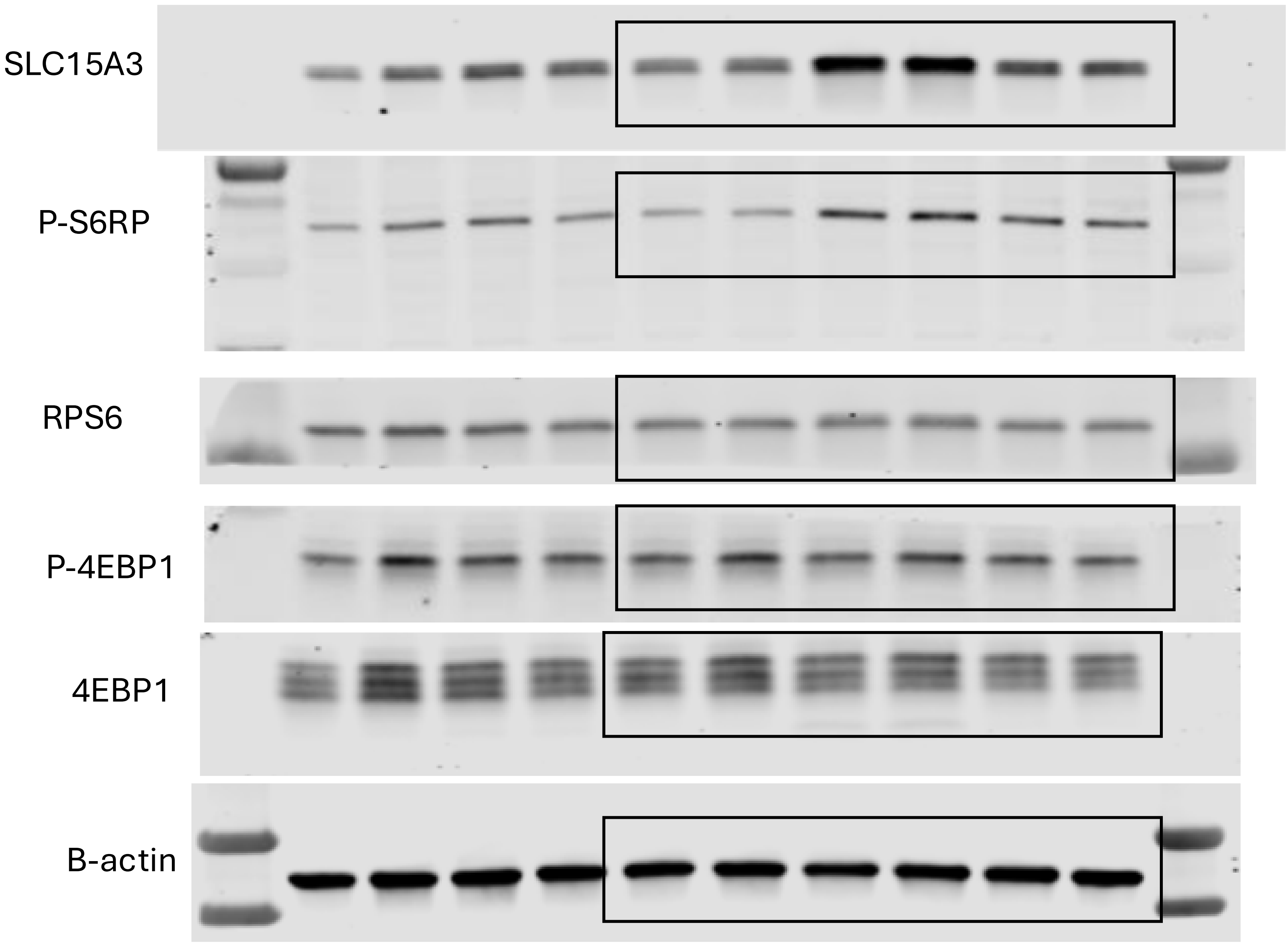

Figure 3j

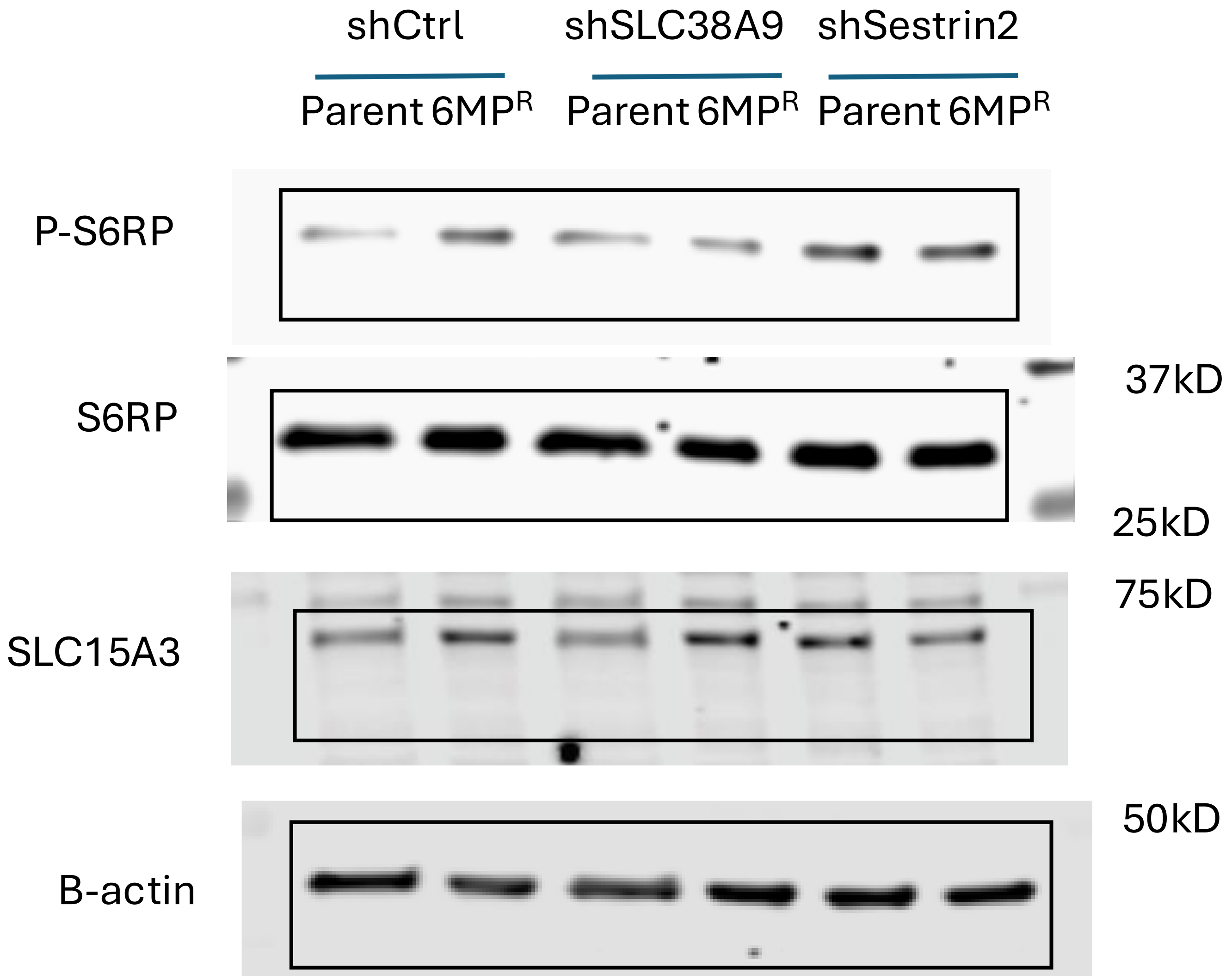
shCtrl
shSLC38A9
shSestrin2
Parent 6MP^R
Parent 6MP^R
Parent 6MP^R
P-S6RP
S6RP
37kD
25kD
SLC15A3
75kD
B-actin
50kD

Figure 4f

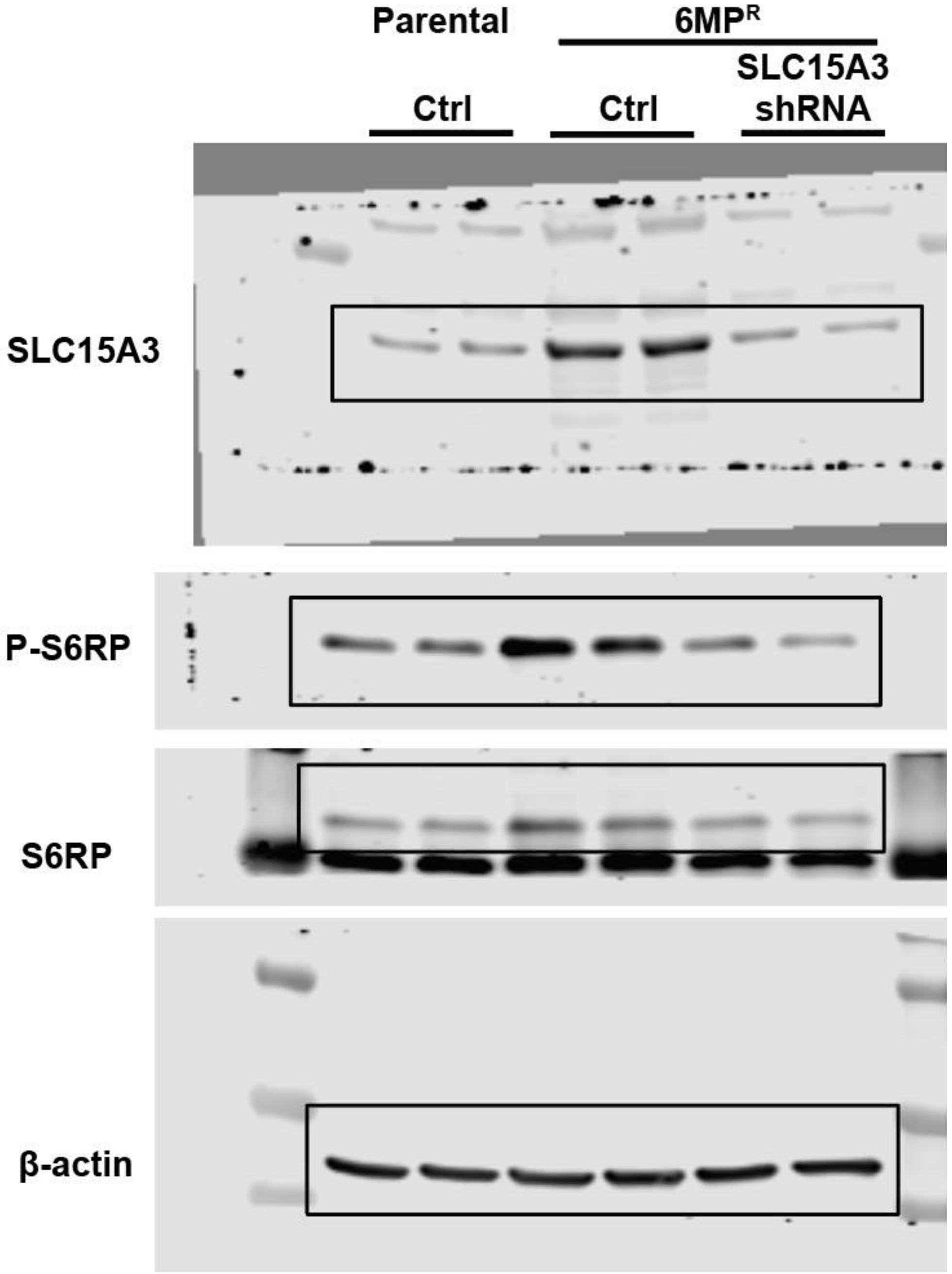

Parental
6MP^R
Ctrl
Ctrl
SLC15A3 shRNA
SLC15A3
P-S6RP
S6RP
β-actin

Supplementary Figure 1d

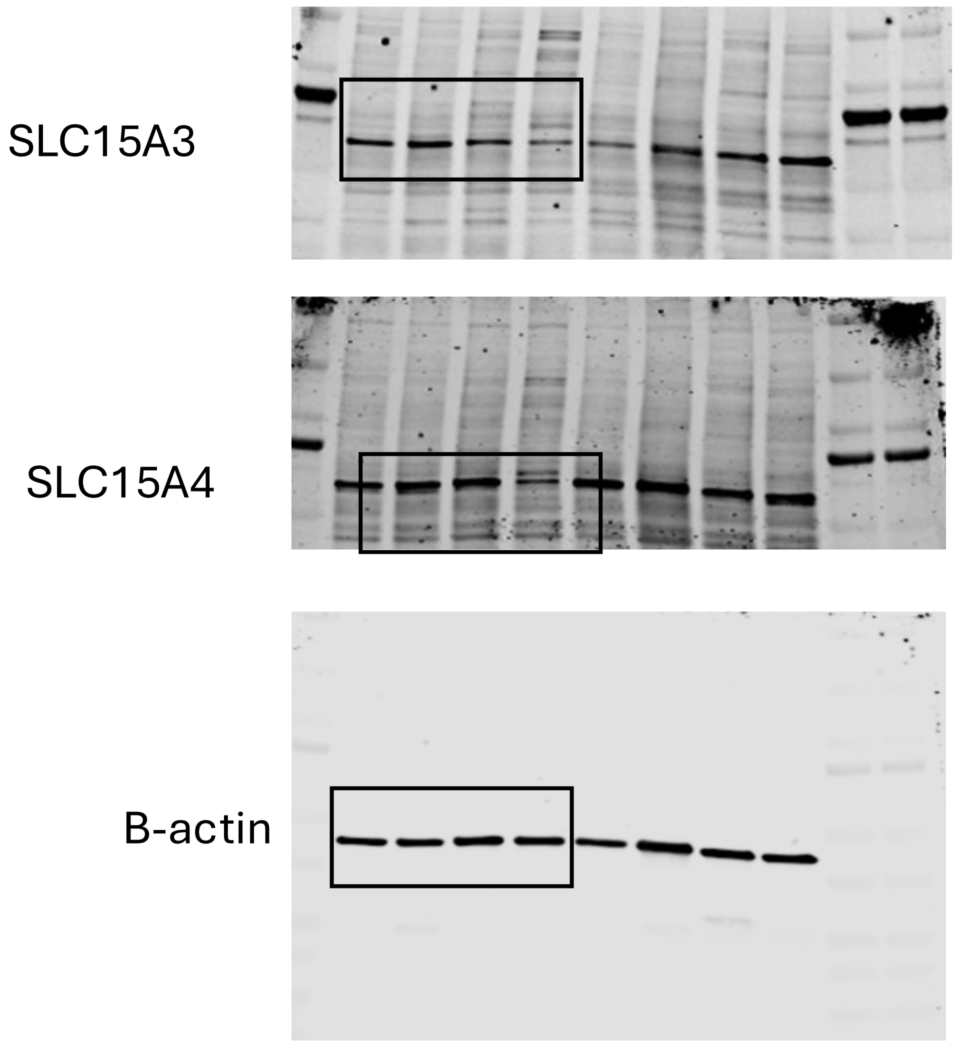

Supplementary Figure 3a

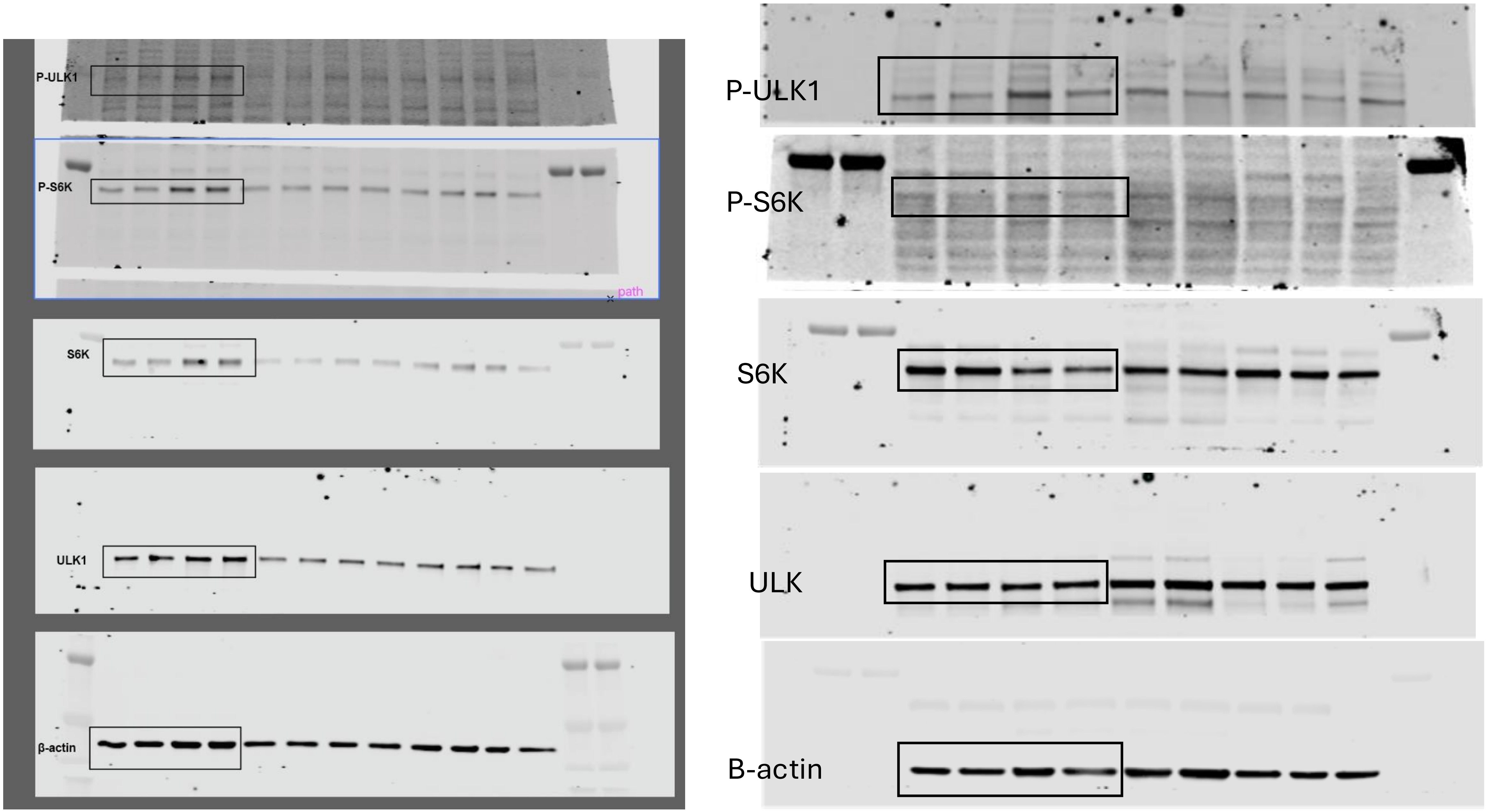

Supplementary Figure 3a

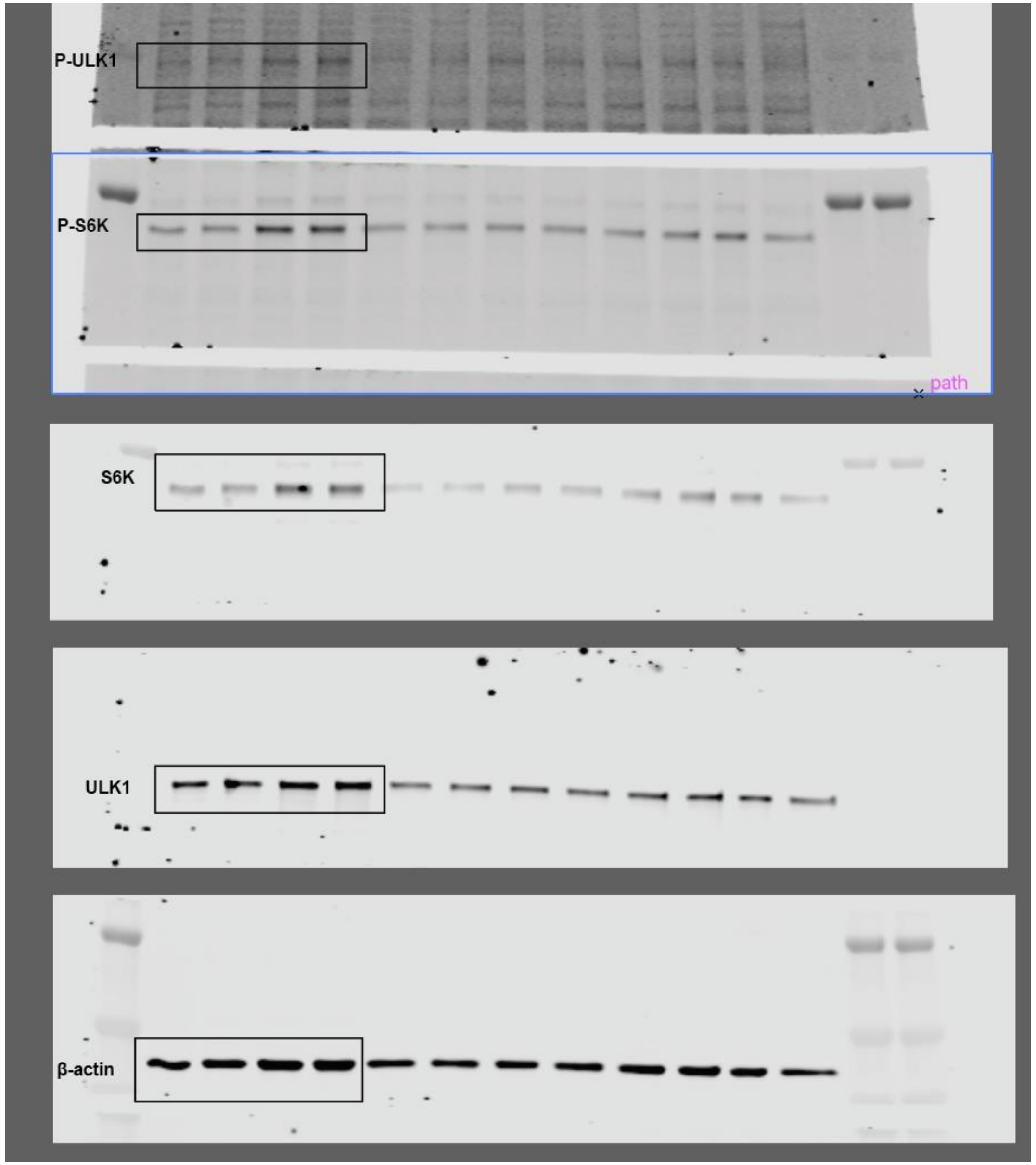

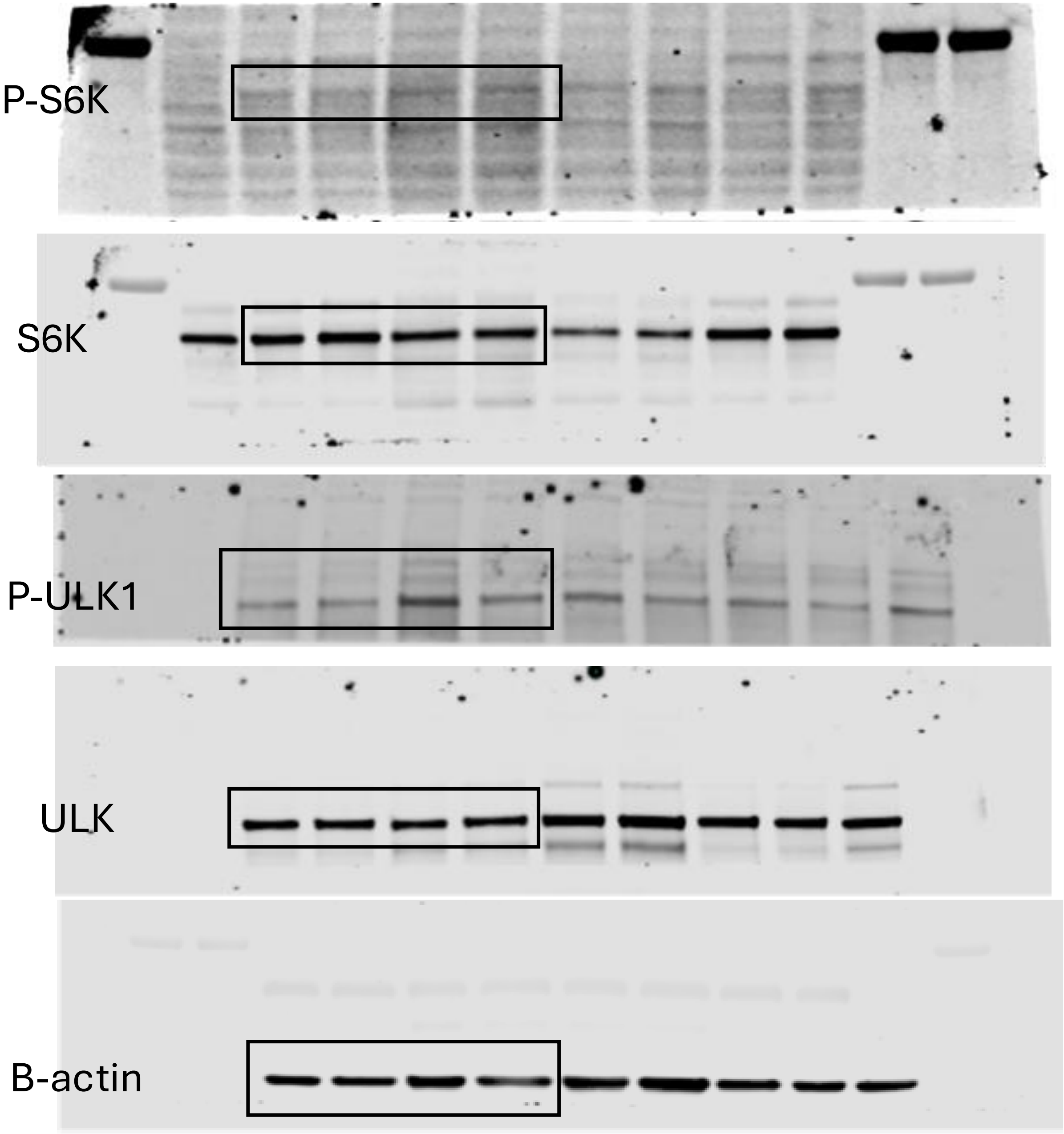

Supplementary Figure 3b

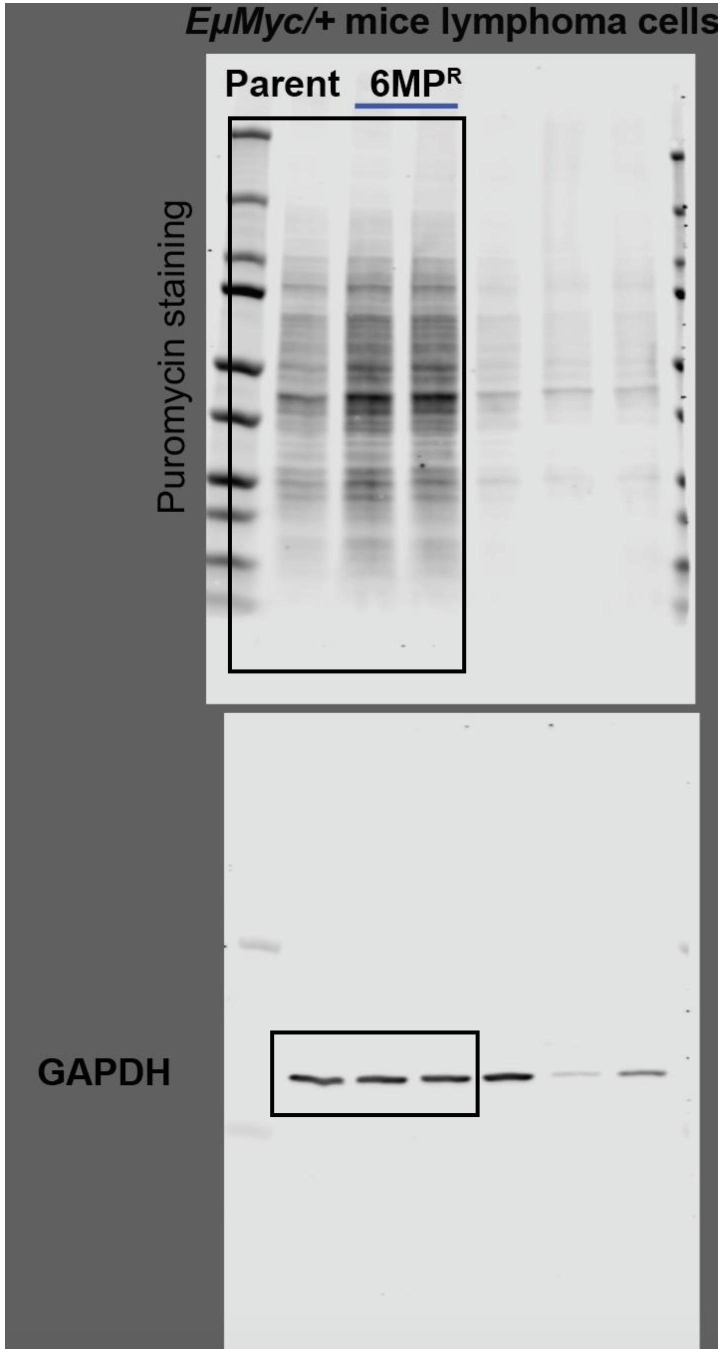

Supplementary Figure 3e

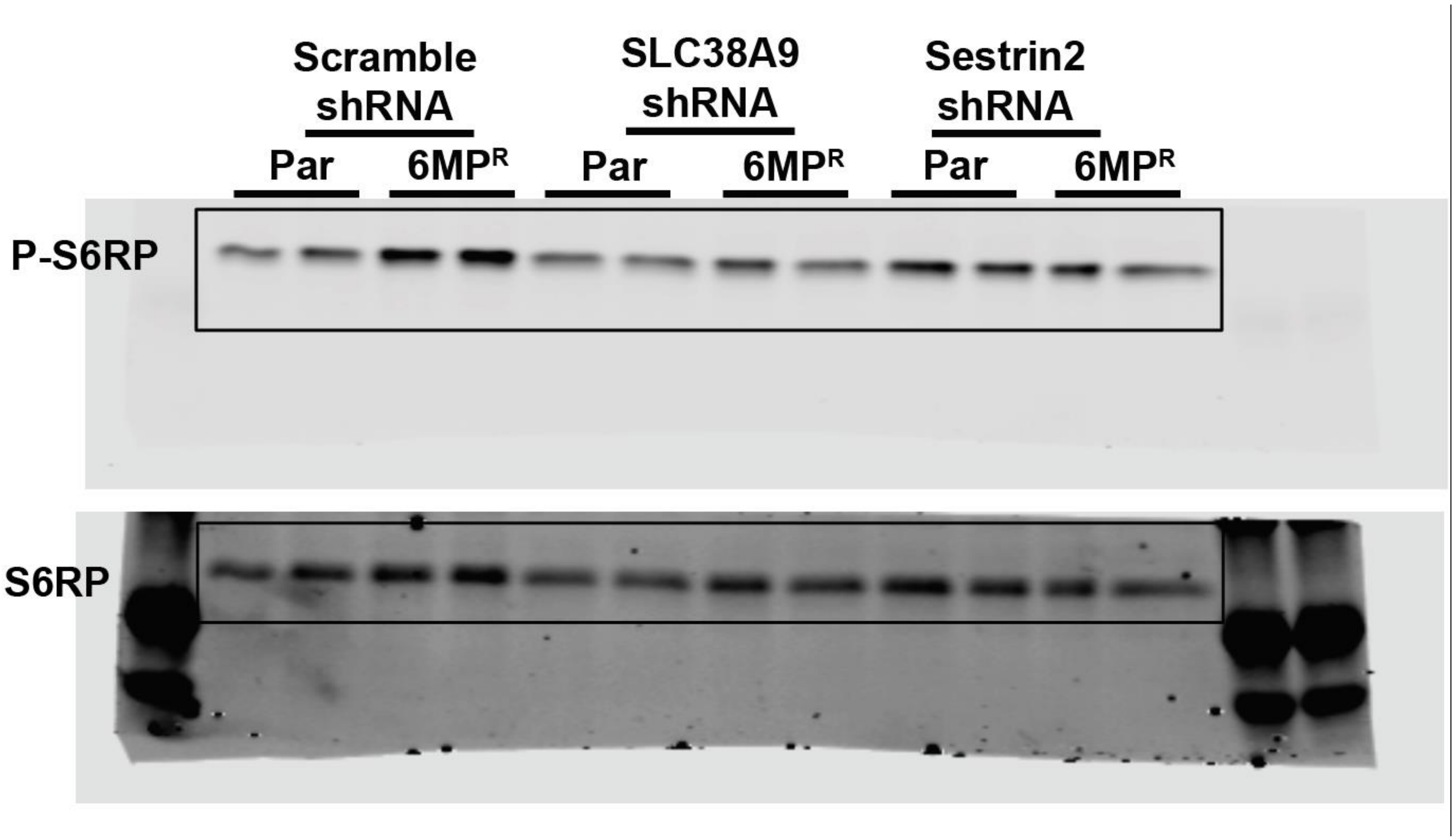

Supplementary Figure 4c

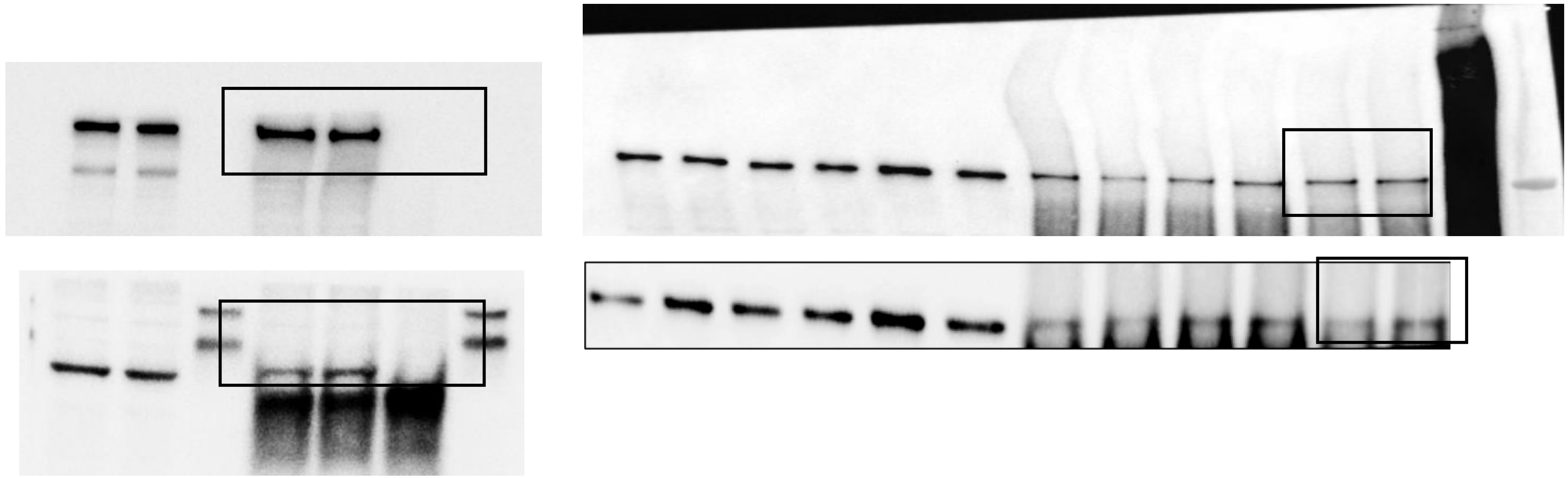

Supplementary Figure 6i

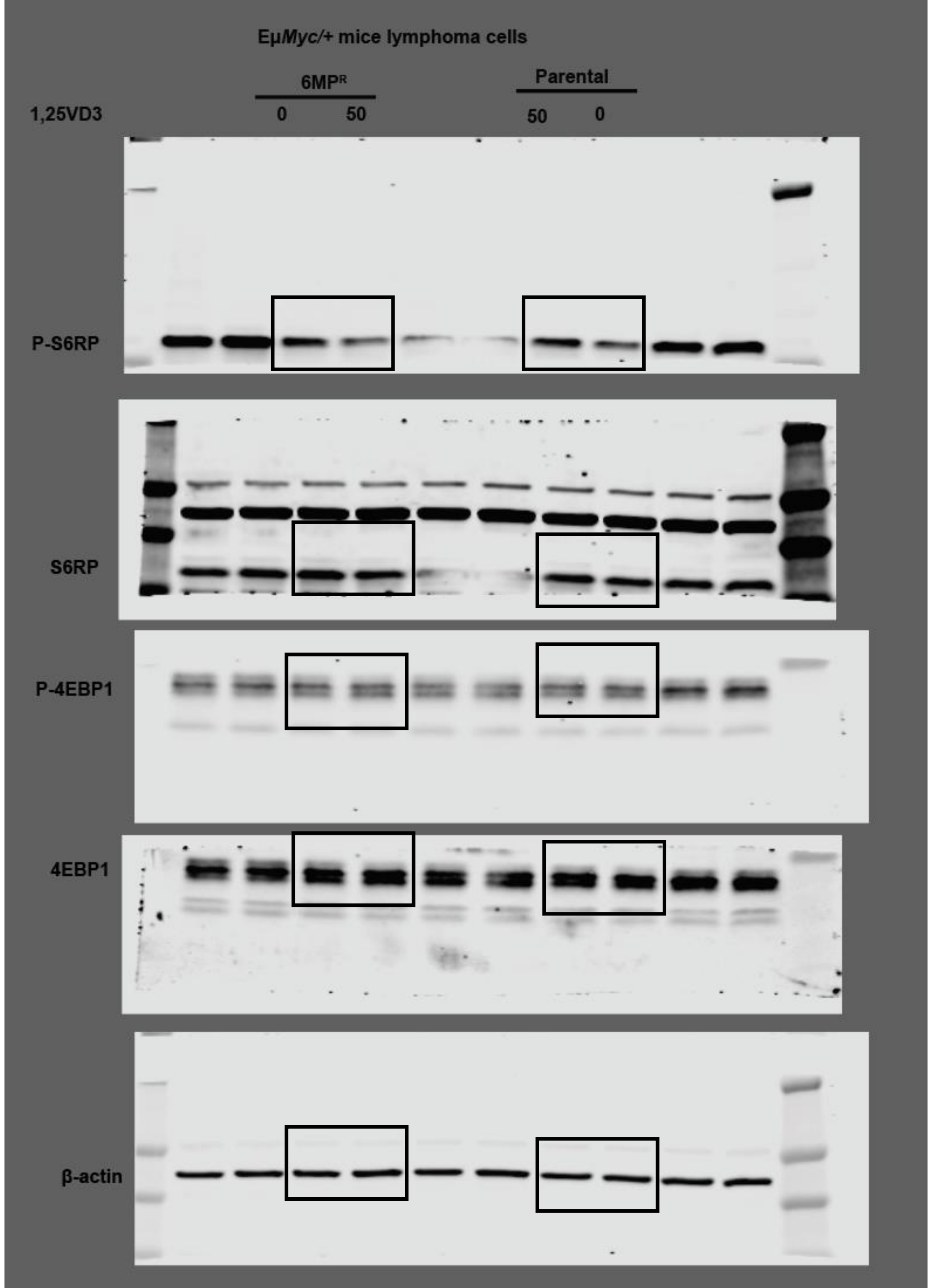

Supplement: Unedited blot and gel images [file jci-136-199709-s041.pdf]
